# Intracranial hemorrhage segmentation and classification framework in computer tomography images using deep learning techniques

**DOI:** 10.1038/s41598-025-01317-3

**Published:** 2025-05-17

**Authors:** S. Nafees Ahmed, P. Prakasam

**Affiliations:** School of Electronics Engineering, Vellore Institute of Technology, Vellore, India

**Keywords:** Intracranial hemorrhage, MUNET, Deep learning, Segmentation, Classification, Computational biology and bioinformatics, Engineering

## Abstract

By helping the neurosurgeon create treatment strategies that increase the survival rate, automotive diagnosis and CT (Computed Tomography) hemorrhage segmentation (CT) could be beneficial. Owing to the significance of medical image segmentation and the difficulties in carrying out human operations, a wide variety of automated techniques for this purpose have been developed, with a primary focus on particular image modalities. In this paper, MUNet (Multiclass-UNet) based Intracranial Hemorrhage Segmentation and Classification Framework (IHSNet) is proposed to successfully segment multiple kinds of hemorrhages while the fully connected layers help in classifying the type of hemorrhages.The segmentation accuracy rates for hemorrhages are 98.53% with classification accuracy stands at 98.71% when using the suggested approach. There is potential for this suggested approach to be expanded in the future to handle further medical picture segmentation issues. Intraventricular hemorrhage (IVH), Epidural hemorrhage (EDH), Intraparenchymal hemorrhage (IPH), Subdural hemorrhage (SDH), Subarachnoid hemorrhage (SAH) are the subtypes involved in intracranial hemorrhage (ICH) whose DICE coefficients are 0.77, 0.84, 0.64, 0.80, and 0.92 respectively.The proposed method has great deal of clinical application potential for computer-aided diagnostics, which can be expanded in the future to handle further medical picture segmentation and to tackle with the involved issues.

## Introduction

Intracranial Hemorrhage (ICH) is a clinically hazardous medical lesion with a high death rate. Leakage in the blood vessels of the brain causes intracranial hemorrhage disease, which results in inactive body functions like speech, vision, memory loss, paralysis and even death^1^. ICH poses an important health issue since it is a large global contributor to morbidity and mortality^2^. There has been a steady increase in the number of ICH cases during the last few decades^3^. Because of the increased risk of death and morbidity associated with ICH, the impact on healthcare systems and economies is increasing^4,5^. An estimated 3.44 million incidents, 3.02 million fatalities and 79.4 million disabilities recorded in 2021 were linked to ICH, according to statistics from the Global Burden of Disease (GBD) research. Between 1990 and 2021, the number of people with disabilities surged by 25.73%, the death rate increased by 41.29%, and the incidence of ICH soared by 46.05% worldwide^6^. Foreman et al. predicted that in the years 2016–2040, the global number of stroke deaths would increase by 8%, with an expected 6.6% increase in deaths from ICH, which would jump from 2.84 million to 3.03 million^7^.

Radiologists manually inspecting CT scans are the current norm for ICH evaluation. Unfortunately, this method is prone to errors due to its vast number of scans and the likelihood of misinterpretation. Early detection and treatment of ICH are crucial in order to enhance the results while easing the burden of this potentially fatal medical condition^8^. In order to help physicians detect cerebral bleeding lesions from CT scans in an effective, dependable, and timely manner, automated models are being developed^9^. The surge in processing speed of computer-aided diagnostic (CAD) approaches has made them preferred techniques for manual assessment in CT image analysis.

Improved patient outcomes and more precise diagnoses could result from the creation of CAD systems, which have the potential to completely transform medical imaging analysis. These tools demonstrate the progress that has been made in this area by helping physicians diagnose patients more quickly and accurately. However, a significant percentage of patients (more than 10,000 annually) still pass away within seven days following their discharge from the urgent care facility without having a diagnosis of a condition that is life-limiting^10^. This is true even with the availability of contemporary imaging technologies.

**Fig. 1:**
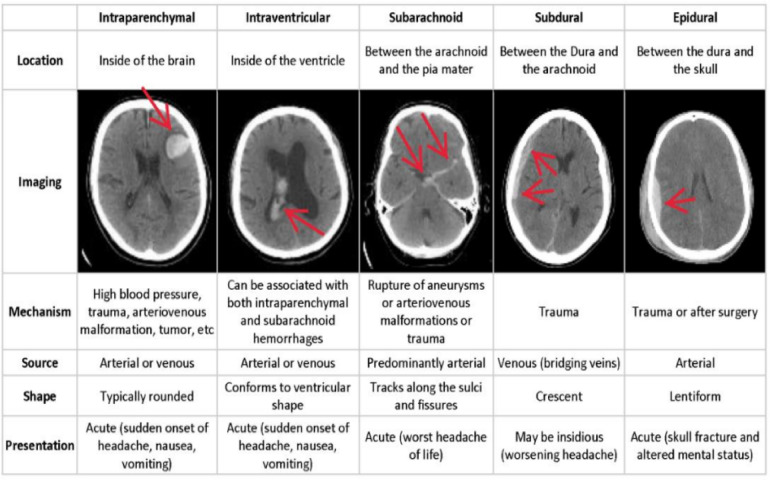
Various types of hemorrhages and their location inside the skull^12^.

SAH, EDH, IVH, IPH and SDH are the five subtypes of intracranial hemorrhage (ICH) that are distinguished by their location in the brain. Damage to the skull, dura matter causes epidural hemorrhage. Blood pools in the tissues of brain are known as intraparenchymal hemorrhage, whereas bleeding into the ventricular system of brain is known as intraventricular bleeding. When blood collects beneath the pia mater and the arachnoid, the subarachnoid emerges. Blood flowing between the arachnoid and dura maters induces subdural bleeding^11^. Figure 1 shows several types of hemorrhage impact in multiple areas of the skull.

When evaluating ICH patients in an emergency, the scan using CT is frequently utilized. For the preliminary evaluation of ICH, CT scanning is a recommended diagnostic method over magnetic resonance imaging, due to its rapid acquisition time and ease of use. Computed tomography (CT) yields the most precise details about thinner tissue with fewer distortions. CT scan uses X-ray beams and varying intensities to capture the brain tissues according to the tissue’s X-ray absorbency then produce a series of images.

A windowing technique can be used to display CT scans in accordance with the level of window, width parameters and transforms to grayscale values. The grayscale image displays various elements of the brain tissues based on the window settings that are selected^13^. The ICH regions show up as hemorrhagic regions with a comparatively ill-defined CT scan’s structural, which are shown utilizing the brain window. An expert radiologist evaluates the CT scans and identifies the location and nature of the illness. By fragmenting an entire brain CT scan into smaller pieces, the major goal is to distinguish between the region and healthy brain tissue. In order to correctly diagnose ICH, create treatment plans, and track their progress, precise segmentation is essential. Variations in hemorrhage patterns, sizes, and locations, along with noise and anomalies in CT images, make segmentation challenging. In addition to lowering the chance of consequences including brain damage and disability, early therapy can assist to improve the outcome. In the field of medical image segmentation, deep learning models such as U-Net and its variations attention-based and transformer-based networks have lately made significant advancements. In the same manner, deep learning has offered reliable technical assistance for ICH segmentation^14^.

A severe medical illness known as intracranial hemorrhage (ICH) can result in life-threatening complications or even death, where early detection and treatment are essential. The main diagnostic method for ICH is a CT scan, which can be laborious and prone to mistakes. To address this issue, deep learning models like U-Net and its variations are created to automate the segmentation and detection of hemorrhagic areas. These novel techniques improve the precision and effectiveness of identifying the hemorrhagic regions and supports early diagnosis and therapy planning. The performance shows the potential for enhancing patient outcomes and developing advanced medical imaging technology.

### Motivation

ICH can happen in several areas of the brain with unclear borders and varies widely in shape. Motion artifacts in CT images typically result in decreased segmentation performance. Various radiologists may identify various hemorrhagic locations inconsistently, and some hemorrhages have ill-defined boundaries. Scientists have proposed a number of solutions to address these issues, such as deformable models, region amplification, and density threshold processing. Nevertheless, these techniques are confined to a small feature representation and subpar generalization performance because they rely on expert-made feature extraction. When using traditional techniques, smaller datasets are used to detect a small number of bleeding classes, and some of these techniques are computationally costly, initialization-sensitive, and needs several iterations to reach convergence. The majority concentrated on categorizing or grouping specific hemorrhage types.

There aren’t many studies that categorize and segment the five different forms of bleeding. Hemorrhage detection is very difficult and laborious procedure that even experienced radiologists find difficult to complete due to the noise, artifacts and comparable regions of pixel intensity in the CT images^15^. Besides, manual evaluation and estimation tasks are highly dependent on the operator and vulnerable to variation in the observers. This kind of labour-intensive process could also lead to mistakes and delays in a large-scale healthcare setting. Furthermore, in the absence of qualified specialists, especially at odd hours, a number of cases of clinical and incorrect diagnosis consequences have been recorded^16^.

The hemorrhagic areas can look diverse in sizes, shapes, and positions. Consequently, the automated identification procedure presents a specific challenge, particularly in cases where the hematoma is small. The study’s primary objective is to classify and separate different hemorrhagic patterns in CT scans with skewed structures; however, the more intricate the network, training will become more challenging and labor intensive.

A network for deep learning network is overly simplistic will have lower detection accuracy because it will miss key hematoma features^17^. In medical imaging, the deep learning techniques may automatically identify and segment specified regions, eliminating the human need and greatly increasing segmentation efficiency by lowering the possibility of manual error but more consistency in segmenting the medical image’s marginal details and small features are required than in natural images. We introduce MUNet, a feature pyramid-based framework for image segmentation designed to address these challenges and meet the growing demand for more precise medical image segmentation.

### Research contributions

The main contributions of the proposed research are as follows.

- Proposed an IHSNet framework for segmentation and classification of ICH in CT images using a deep learning model.

1. The proposed framework incorporates various pre-processing methods such as resizing, CLAHE, etc., to improve the interpretation, evaluation for the diagnosis of bleeding disorder CT scans.
2. Applied the SMOTE based technique for solving class imbalance in order to tackle with the issues of minority class imbalance present in the dataset.
3. Proposed a multiclass UNet (MUNet) model for segmenting and classifying the various ICHs in brain through CT images which may aid the healthcare professionals to diagnose the ICH regions in an accurate way.

- Proposed framework successively obtains enriched high-quality feature maps from the encoder then merged with correspondingly rich semantic feature maps from the other sub-network (decoder).
- Includes a feature pyramid network in the proposed model to enable it attains faster and more effective detection. The structures of various hemorrhages can be segmented and finally detailed features can be captured.
- Propose a multiclass classifier along with MUNet where the components CBL layers and convolution layer along with fully connected layers to classify intracranial hemorrhages.
- Evaluate the performance of the proposed IHSNet framework through Dice Coefficient, IoU for segmentation and Precision, Recall, F1 score, AUC-RoC and classification accuracy for classification.
- Compare the performance with existing methods to claim the superiority of the proposed IHSNet framework.

## Related works

Segmentation has greater medical relevance in a study of an ICH image analysis since it precisely recognizes the bleeding location in the various regions of the brain. Several segmentation techniques have been used to assess medical diagnostic images.

In 1943, McCulloch and Pitts made artificial neurons first known^18^. Later on, as technology and systems advanced, this approach was refined and used as a trailblazing method for image analysis across a range of industries. Yuh et al. created a threshold-based approach to identify ICH in contrast to the conventional machine learning techniques. The technique used to identify the ICH subtypes was based on their location, volume, and shape^19^. Using retrospective samples from 33 CT scans, the investigators improved the threshold value and assessed their model on 210 CT scans of patients who might have traumatic brain injury. Their approach produced ICH detection results with 98% sensitivity, 59% specificity, and moderate accuracy for ICH sub-type detection.

The segmentation models with a modified encoder-decoder architecture based on the U-Net are the most popular ones regarding deep learning. UNet^20^ utilizes a encoder-decoder architecture (symmetric) with a skip connection and shows exceptional performance in the segmentation of medical pictures by multiscale features convergence. The segmentation model was proposed in 2015. Due to their superior performance in medical images analysis, several models shaped by U-Net and based on U-Net, namely 3D U-Net, Attention U-Net and U-Net++^21^, been utilized widely. In addition, a lot of people have embraced encoder-decoder architecture models like SegNet and PSPNet. Owing to the U-Net model’s effective and straightforward architecture, the majority of advancements in biomedical image segmentation are based on it. Still, there are certain limitations that restrict its potential. There have been many advanced frameworks developed recently to enhance their effectiveness in healthcare.

Zhou et al.^22^ suggested U-Net + + for segmentation of polyps, brain tumors, liver then cell nuclei from various modalities, and it has shown promising results. U-Net + + is an encoder-decoder sub-networks accompanied by dense skip links and stacked sub-networks to extract multi-scale features^23^.The main weakness in U-Net++, in spite of its higher performance, is the amount of work and complexity it requires to execute an extensive number of intermediary convolution operations. The U-Net3 + design, a full-scale connected U-Net with deep supervision that increases the utilization of feature maps in full sizes, was also introduced by Humin et al.^24^.

Based on hyper column features and void convolutional neural networks, Islam et al.^25^ proposed ICHNet in order to divide the lesion region, Kuang et al.^26^ introduced the Semi-D-Unet model, which included weighted cross entropy for three models. This results in a high complexity in model with sluggish training pace. Kuo et al.^27^ suggested a neural network design for concurrent segmentation and tasks for classification. Using Dilated ResNet38 as the framework, they created branching for block classification and pixel prediction by adding up the variations in the acquired image characteristics from each CT scan separately.

The asymmetry of the ICH healthy brain tissue was the main focus of Liang et al.‘s study^28^. They segmented the hematoma site using attention-boosting symmetry in a U-shaped network structure and converted the cranial CT scans into the normal site of bilateral brain tissue symmetry. Chang et al.^29^ suggested using a mask RCN trunk to identify and separate three types of cerebral bleeding: epidural and subdural blood, subarachnoid hemorrhage, and cerebral hemorrhage. This method recovers the image resolution using a 2D extended network structure after extracting features using a 3D pyramid network path. This method needs a high operating resource and huge number of operations with 3D convolution because it uses a 3D network. Cho et al.^30^ suggested five distinct types of cerebral hemorrhages—ventricular, subarachnoid, epidural, and parenchymal were segmented using Dual FCNs in order to balance sensitivity and specificity. In 2020, LcGAN^19^ introduced an optimised segmentation model to enhance data quality and augment learning. The aforementioned methods primarily rely on convolutional neural networks, which possess adequate capacity for extracting local features. However, they fail to account for the variety of clinical data and do not make both high and low-level semantic information, leading to a significant loss of extracted image information from the network.

### Classification

The two objectives of research on ICH subtype categorization are to identify whether ICH is present in a CT sequence and to classify IPH, EDH, IVH, SAH and SDH. Physicians can diagnose patients with ICH with the use of research, and they can locate abnormalities more quickly owing to the five-subtype classification. Deep learning and classical machine learning-based approaches are the two main categories of current research on the classification of ICH subtypes. Manually created features are a common component of traditional machine learning techniques, and creating these features requires sophisticated background knowledge in medicine. The quality of the features retrieved in this way, which are also known as handcrafted features, directly influences how well the classification system performs.

Yuh et al.^32^ created a threshold-based system for ICH detection based on the concept of classical machine learning. The algorithm categorized the hemorrhage’s position, shape, and volume into different subtypes. A convex optimization function was solved by Chung et al.^31^ using a continuous max-flow technique. Medical specialists must manually create features using these methods, which rely on prior understanding of ICH. Feature extraction in conventional machine learning techniques necessitates not only highly qualified researchers but also a significant amount of labour and material resources.

Deep learning, on the other hand, extracts feature very deeply utilizing a CNN and a substantial quantity of training data, negating the need for human feature extraction algorithm creation. Phong et al.‘s study was among the first to use deep learning for ICH identification^33^. Researchers have discovered that diagnosing ICH can be accomplished with good results using a pretrained CNN. The likelihood of each subtype in a CT slice was predicted by Chilamkurthy et al.^34^ using the ResNet network. The random forest was then fed the output layer result for each slice to determine the subtype prediction and hemorrhage for the entire CT sequence. A 3D CNN^35^ approach for ICH tasks were presented by Arbabshirani et al.^36^. In the discipline of ICH classification, Kuo et al.^37^ proposed the idea of active learning and employed unlabelled training data to enhance deep learning network performance.

In a work presented in^38^, ICH and its types are classified on CT images using deep learning. At the Russian Burdenko Neurosurgery Center, real-time analysis of CT image is done using deep neural network (DNN). The optimizer (adam) adaptively modifies the channel-wise feature responses, is used in conjunction with the ResNext50 architecture to achieve precise categorization. IVH is categorized with an accuracy of 89.3% in this work. Setting and generalizing hyperparameters such as stride and filter size for real-time datasets can be difficult.

Ensembled deep neural networks are introduced in^39^ for the diagnosis of ICH. Using a hybrid category-weighted loss function and a patch-based balanced sampling approach, Clerigues et al.^40^ developed and assessed an encoder-decoder based structured network (asymmetric) that combined short and lengthy residual connections to address the issue of category imbalance. A CT slice undergoes preprocessing for five distinct types (IPH, IVH, SAH, SDH, and EDH) and two categorization types (ICH and non-ICH). Grad-CAM is utilized for this reason in addition to accurately identifying bleeding. Every ICH subtype is examined using an automated method that operates at the slice and subject levels. Processing of 3D CT scans is faster than that of 2D CT scans. For every ICH subtype, the AUC value is higher than 0.8.

The authors in^41^ presented by Combining LSTM, 1D-CNN, and logistic regression (LR)models as an ICH radiological report classification system based on natural language processing (NLP). For feature extraction, LSTM and CNN models were employed, and for classification, the LR function was utilized. The model’s AUC of 0.94 indicates severe unreliability for applications involving vital medical information. Real-time CT scans of ICH sufferers and healthy controls were employed by Lee in a study^42^. Three forms of ICH—EDH, SAH and IPH were classified by use of Kim-Monte Carlo-based deep learning algorithms. Achieved accuracy rate of SAH was 91.7% versus an overall 69.6%. The primary shortcoming of this study is that a larger sample size was needed to confirm the findings.

An overview of state-of-art literatures related to ICH segmentation and classification is reviewed in Table 1.

**Table 1:** An overview of state-of-art literatures for ICH segmentation/classification using deep learning techniques.

Authors	Dataset	Segmentation/ Classification	Model	Metrics	Limitations
Xiao et al., (2024)^43^	ICH-Seg, Physionet	Segmentation	MPFR-Net	Dice-0.7044, IoU- 0.7042 Precision- 0.7218 Sensitivity-0.7215	Failed to fully utilize the spatial information that existed between slices.
Petrov, et al., (2024)^45^	Almazov National Research Medical Centre, Russia	Segmentation	Computer vision-based method using the U-Net.	Dice-0.77	Did not provide an automatic method/model for the segmentation of hematoma.
Xu et al., (2023)^46^	Tiantan Hospital, China	Segmentation	CHSNet	Dice = 0.918, ASD = 0.476, IoU = 0.853	Label training is required for the built neural network model.
Wang et al., (2023)^47^	Cooperative hospital	Segmentation	GroupCapsNet grouped capsule network	Dice = 0.87, IoU = 0.76	The developed network consumes very high memory and abundant computation.
Yu et al., (2022)^44^	Xuzhou Central Hospital, Xuzhou, China	Segmentation	DR-UNet	Dice- 0.874 ± 0.130, R2 = 0.9979, *P* < 0.0001	Since all ICH patients were gathered from a single institution, the scope of DR-UNet requires further validation
Akram et al., (2023)^48^	RSNA-2019	Classification	DBXA	Specificity-0.938, Precision-0.970, F1 Score-0.953	This study’s data set involves noisy data.
Yuan et al., (2022)^49^	MICCAI 2020 ADAM	Segmentation	Dense convolutional attention U-Net	Sensitivity-0.788, Precision-0.788, Dice score-0.745	The method cannot be generalized to other imaging methods and scanners.
Liu et al., (2023)^50^	RSNA-2019	Classification	CNN-RNN architecture	Recall-0.7916, Precision-0.8629, F1 Score-0.8248	Numerous hyperparameters in the model require optimization.
Umapathy et al., (2023)^54^	RSNA-2019, CQ500	Classification	SE-ResNeXT and LSTM	Recall-0.98, Precision-0.96, F1 Score-0.97	Due to training time limits, it was challenging to perform more analysis and difficulty in understanding how the pipeline operates.
Hussain et al., (2021)^51^	RSNA-2019	Classification	ResNet152V2 + Attention	Sensitivity-0.971, Specificity-0.978, F1 Score-0.961	Since additional GAN versions might produce even better results, only a DCGAN-based framework for augmenting data is tested.
Alfaer et al., (2021)^53^	Physionet	Classification	AICH-FDLSI	Sensitivity-0.8861, Specificity-0.9833	Hybrid DL models could have been implemented to enhance the AICH-FDLSI approach.
Barin et al., (2021)^55^	RSNA, Kaggle	Classification	Hybrid architectures ResNet Inception-V2 and EfficientNet-B3	Accuracy-0.985	There are numerous images without hemorrhage than the epidural type, this could have impacted on low sensitivity.
Ye et al., 2019^52^	The First Affiliated Hospital of Shenzhen University, China	Classification	Multi-stage AI system with RNN and 2D CNN.	Sensitivity-0.95, Accuracy-0.988 Specificity-0.944, AUC-0.8	Further investigation required to address low SAH identification, which limits the performance.

### Problem identification

- The majority concentrated on categorizing or segmenting specific hemorrhage subtypes. For the five hemorrhage subtypes, few studies have been carried for both segmentation and classification.
- Conventional machine learning methods are costly to compute, responsive to initialization, and necessitate maximum iterations before reaching convergence.
- The application’s generalizability is diminished since for certain ICH subtypes, the data set size is constrained.
- Blurred borders, smaller features and texture problems pose obstacles for automated segmentation, affecting its accuracy.
- The precise diagnosis of ICH can be difficult for radiologists to make from CT scans because **ICH** regions can be mistaken for calcifications or stripping artifacts.
- More precise segmentation is required for the medical image’s tiny features and marginal details compared to natural images.

## Materials & methods

**Fig. 2:**
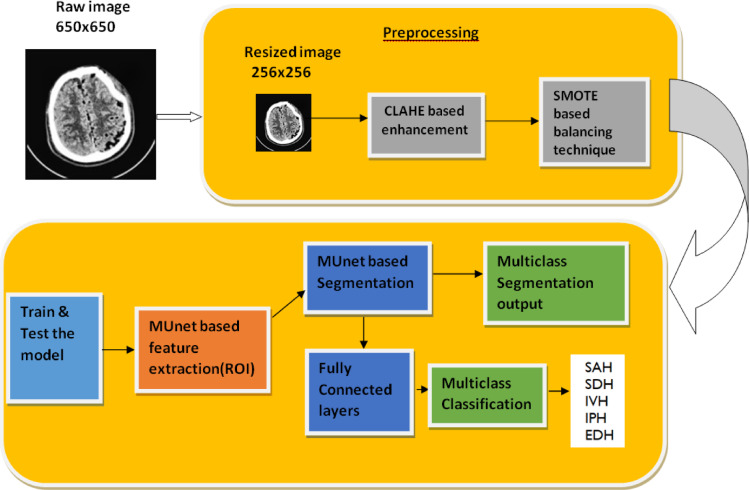
Framework of the proposed model IHSNet.

The study’s details are described in this section, and Fig. 2 shows the suggested framework for segmentation and classification.

### Dataset

This study used Physionet public dataset. The public dataset, which was collected at a hospital named Al Hilla in Iraq, comprised the CT scans of 82 patients (46 were men and 36 were women) with brain injury, with the mean age of 27.8 ± 19.5 years. Of these patients, 36 had intracranial hemorrhage. This dataset is feasible even with an age up to 48 years. Other research proposed segmentation methods for ICH in addition to ICH classification and detection. However, most of these techniques were not verified because of the absence of their corresponding ICH masks, which is why these discrepancies exist, and it is not possible to conduct an impartial evaluation of the different approaches. Consequently, a dataset that can support benchmarking with expansion is needed. In our study, PhysioNet dataset is used, as it offers expert-annotated bleeding masks which are crucial for assessing the segmentation performance. Using ICH segmentation and the corresponding masks that are available in Physionet, the main objective of this study was fulfilled.

The Siemens/SOMATOM CT scanner with the specification was given in Table 2.

**Table 2:** CT scanner specifications.

Parameter	Specification
Scanner	Siemens
Tube voltage	100kv
Tube current	28-500 mA
Dynamic range	102 db
Isotropic resolution	0.33 mm
Slice thickness	5 mm
Slice interval	5 mm

The slices count in a CT scan is roughly 30. Of the 82 individuals, 36 had an ICH of any of the following types: IVH, EDH, IPH, SDH and SAH. Three hundred and eighteen CT slices with ICH in the dataset were used for testing and training, since the study omitted the number of slices that did not have an ICH. It is noteworthy that the dataset exhibits an imbalance in the CT slices counted for each sub-type of ICH, with numerous of CT slices without an ICH. During the initial processing, every CT slice was stored as a 650 × 650 grayscale image. Next, additional preprocessing of the data was done, and it was downsized to 256 by 256 so that it could be utilized for experiments. Patient wise scans were considered in our study to avoid any leakage of similar samples in training and testing data. In the Table 3, the details of patients with type of hemorrhage and corresponding patient id have been included.

**Table 3:** Details of patients ID wise dataset used in the study.

S.no	Hemorrhage type	Patient ID used for training	Patient ID used for testing
1	IVH	85,91,92,94	80
2	IPH	51,58,69,72	50,79
3	SAH	76,82,84	70
4	SDH	71,74,81	51
5	EDH	52,53,66,67,68,73,75,77,78,83,86,93,97	49,87,88,89

### Data preprocessing

Data preprocessing techniques can help an image look richer and shows additional details about the object of interest. The CT images in our collection are pre-processed so that the deep learning network can more easily adjust to the training phase. Images included in our dataset differ in size and form from the deep learning’s input images expects to be of the same size, hence the images of dataset are transformed into a specific size. Each CT slice has its size reduced from its original dimension in order to minimize the usage of computer resources. Addressing skewness of data in for testing, validation and training is also an important goal of data pre-processing. In order to accomplish this, every data for testing, validation, and training dataset needs to have a substantial representation of every type of class inside the data that requires trained.

The sequencing of the combined dataset and rearranging is not directly controllable when all the photos are combined into an array and then divided into training, validation, and testing datasets. This makes the approach inefficient. Thus, prior to merging the datasets, each class has to be splitted into its own training, and testing dataset. Furthermore, overlapping borders, noise, poor contrast, and variations in axial rotation are all problems with CT scans that make them challenging to use. We therefore chose CLAHE over alternative denoising methods in order to detect the hemorrhagic patterns of dural hemorrhages. A better way for addressing class imbalance issues is the SMOTE method which used to balance the unbalanced CLAHE-enhanced images. It preserves texture and spatial information—two factors that are particularly important in dural hemorrhages. SMOTE is used in this study to produce balanced synthetic samples^52^.

#### Resizing

It is important to downscale the photos carefully as part of the data preprocessing stage by looking at the dataset since it may result in loss of some information. Additionally, the GPU RAM may be exceeded when resizing the image to a very big size, therefore the optimal image size based on the experiments is preferred in order to maintain efficient memory and avoid losing any important image information. The major concern during resizing is what a standard size is essential to resize entire images. We can select the most prominent image size and resize all the images to that size, or we can select the smallest image size and resize all the images larger. In order to preserve the aspect ratio of images, the scaled width of 256 pixels will automatically adjust to 256 pixels of height to retain the 1:1 ratio, since the original image had a dimension of 650 × 650 pixels. Without maintaining the aspect ratio, the width and height will be adjusted separately, which may result in the image stretching or changing the originality of image. These issues were not observed during the resizing process.

Larger image pixels cause smaller image pixels to stretch during the stretching process. This could make it more challenging for our model to detect crucial characteristics like object borders. Stretching, in the event that the input aspect ratio is irrelevant, can be a great way to make the most of the pixels transmitted to the network. In order to achieve optimal performance, it is imperative to properly pre-process and resize the data, as it plays a significant role in machine learning and deep learning algorithms^57^. The CT images in our collection are pre-processed in order to better adjust to the training phase of the network, thus it’s beneficial to experiment with progressive resizing. Starting with a lesser image size, we analyse the trade-off between computing expense, image size, and accuracy as we enhance the image size. We used a resize scale of 256 × 256 pixels where training time is significantly less and significant computational benefits are achieved. Furthermore, when scaling the images, no overlap cropping technique was utilized and the built-in Python Pillow library function was used for resizing the images.

The computational costs and memory requirements are greatly increased when images of 512 × 512 are used. On the other hand, 256 × 256 reduces the computational cost and provides a compromise between efficiency and accuracy. In order to improve feature visibility and assist with edge enhancement, CLAHE is used to restore the local contrast, which is sometimes diminished by resizing, making subtle regions more difficult to discern. Preprocessing thereby assists the segmentation models and prevents negative impact. The images of CT scans were annotated with a unique label for each hemorrhage type along with their corresponding masks for better assessment. We chose non-overlapping resizing, because overlapping can aid in capturing contextual information but they result in duplication of data as well as computational expense. No overlap cropping is enough when each patch has sufficient context for segmentation, which can make computations simpler.

#### Contrast limited adaptive histogram equalization (CLAHE)

CLAHE abbreviating Contrast Limited Adaptive Histogram Equalization is a popular enhancement technique used to refine and improve the details of the image. While traditional HE tends to attenuate fine details, it can boost an image’s overall contrast. By concentrating on certain regions of the image to bring out features, the Adaptive Histogram Equalization (AHE) algorithm outperforms the HE algorithm. Its handling of the transitions between various blocks, nevertheless, still needs improvement. By limiting contrast augmentation with a threshold and hence to reduce picture noise, the CLAHE algorithm improves on AHE. Additionally, it uses a linear interpolation technique to provide smooth transitions between image blocks, resulting in thorough and efficient image processing. As the CLAHE algorithm skilfully increases image contrast^58^.

**Fig. 3:**
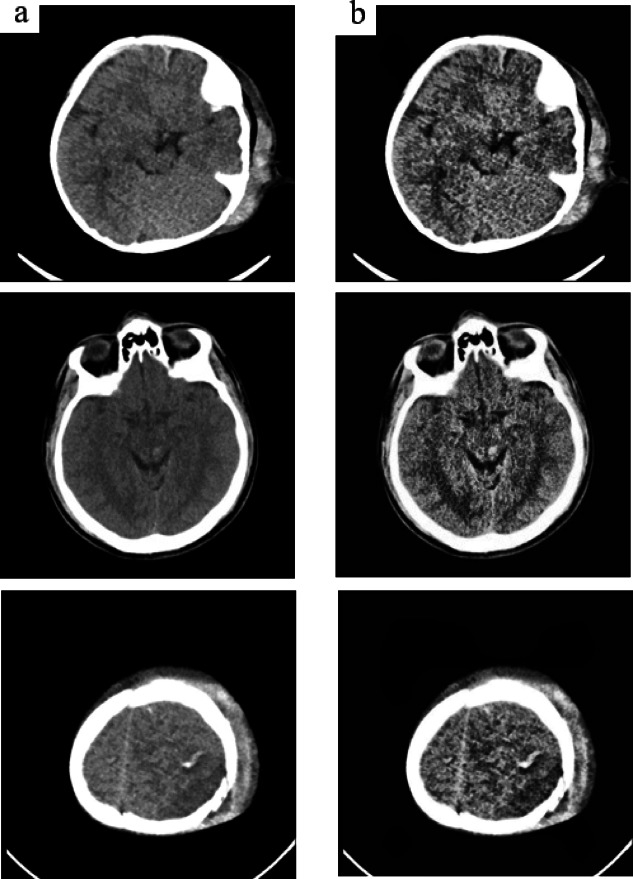
Comparing CLAHE enhancement (**a**) Images before applying CLAHE (**b**) Images after applying CLAHE.

The Fig. 3 depicts the images before and after applying CLAHE enhancement. Since medical images are assigned using intensity levels with the assistance of CLAHE. Limiting contrast intensities in changeable areas, which are identified by histogram peaks in association with temporary regions (i.e., many pixels falling into the same grayscale), may help minimize AHE-related noise concerns.

Slopes associated with CLAHE’s gray level assignment technique are restricted to certain pixel quantities, which are alternatively linked to histograms (local). The histogram is fairly measured by clipping its pixels and maintaining its count equal. Thus, for the image processing tasks including object detection, segmentation, and analysis, CLAHE enhances the appearance of the image and boosts performance. A crisper image and a more accurate computational analysis are the outcomes of image enhancement. Noise reduction, contrast improvement, and image deblurring can be improved with the help of CLAHE^60^.

#### SMOTE-based class balancing

**Fig. 4:**
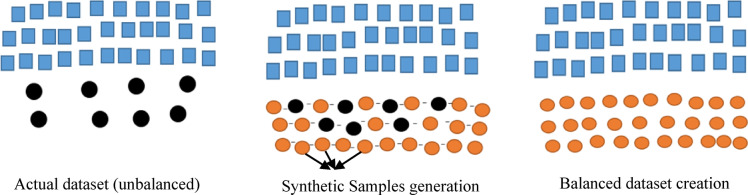
SMOTE Based balancing.

Imbalanced classification difficulties have garnered a lot of attention. The Model’s performance created from datasets that are imbalanced is hard to forecast since it depends on a number of variables, including the extent of the disparity in class, the complexity of the data, the size of the dataset as a whole, and the method of classification used. A circumstance where the distribution of classes is unequal is referred to an imbalanced task, which is different from earlier standard classification issues. This indicates that a certain class usually referred to the more occurrences of the majority class than the other class, sometimes referred to as the minority class. Nonetheless, minority class forecasts often perform worse than majority class predictions in these unbalanced tasks, which results in a significant number of minority class predictions calculated inaccurately. When these discrepancies occur, there are different techniques for balancing datasets. We compared the disparities between balanced and unbalanced datasets in this study using the synthetic minority oversampling technique (SMOTE) and these unbalanced data can be balanced using a preprocessing method called Synthetic Minority Oversampling Technique (SMOTE). The diagram in Fig. 4 depicts the SMOTE based balancing.

SMOTE technique^61^ is a non-destructive one that equalizes the number of samples of each class by generating virtual data points between the minority class’s existing points using linear interpolation. It should be mentioned that there is a trade-off between sensitivity and specificity when utilizing SMOTE for oversampling. An increase in the number of items correctly identified for the minority class is indicated by a more evenly distributed training set. It is an oversampling approach, but rather than copying existing samples, it synthesizes new ones. It provides a balanced dataset and generates samples from underrepresented classes. SMOTE selects the feature vectors from the minority class and then uses Euclidean distance in the feature space to determine their nearest neighbors. After identifying the closest neighbors, the chosen vector and its neighbors are interpolated to create synthetic samples. Random noise is added along the line that connects the feature vector to its neighbour in order to perform interpolation. For a predetermined number of synthetic samples, the procedure is repeated. A random number of neighbors is selected for every sample in the minority class, and new synthetic examples are produced by interpolation. As a result, the minority class is more represented. SMOTE techniques are highly successful in enhancing the ability of the model to generalize since they allow for improved performance with the data (balanced). SMOTE is very useful for producing synthetic samples that mainly retain spatial information, texture, and visual characteristics.

**Fig. 5:**
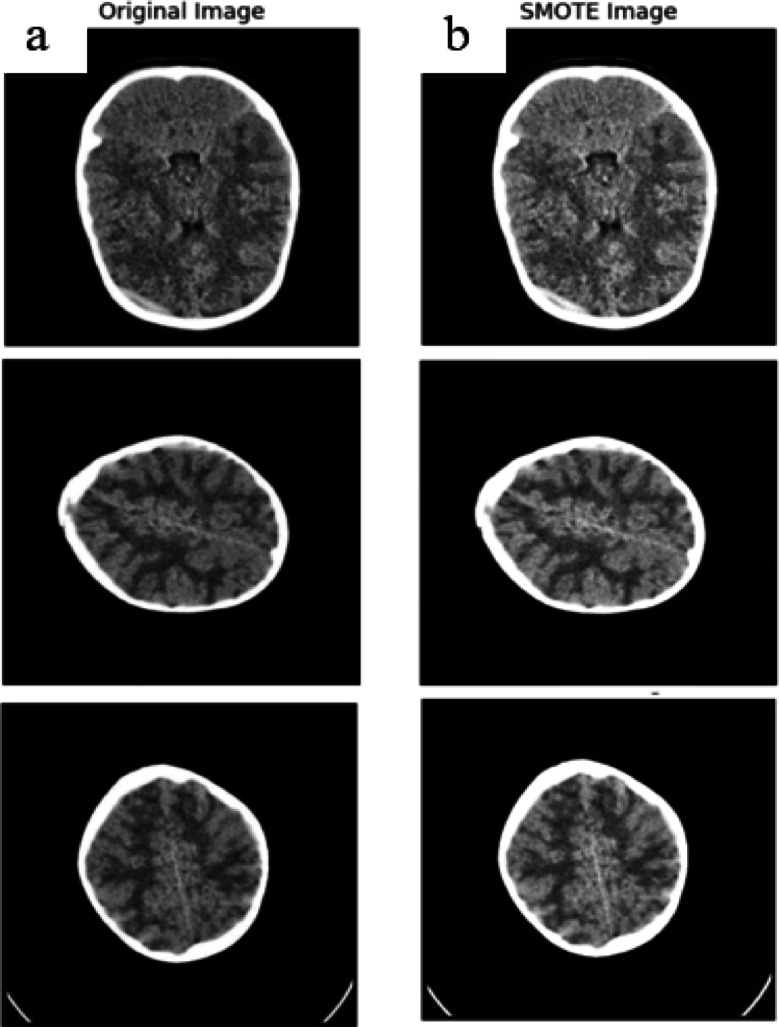
Comparison (**a**) Original images (**b**) SMOTE applied images.

Through implementing this, the model is prevented from overfitting to particular cases in the minority class and redundancy is decreased. In order to produce a more generalized model, SMOTE balances the dataset, lowers the bias, and generates a variety of training samples, throughout which helps avoid overfitting. The original and the SMOTE applied image samples are depicted in Fig. 5.

In our study, we had a total of 2031 images, where 1960 involved in training and 71 were used for testing and no any multi type hemorrhagic occurrence was found within a single image in the Physionet dataset. Number of images before and after applying SMOTE technique is displayed in Table 4.

**Table 4:** ICH images count before & after applying SMOTE technique.

ICH type	Hemorrhagic images (Actual)	Image samples before SMOTE (80%)	Training samples after applying SMOTE	Testing samples (20%)
IVH	24	18	385	6
IPH	73	58	400	15
SAH	18	14	403	4
SDH	56	45	364	11
EDH	173	138	408	35

### Proposed MUNet architecture

The proposed MUNet (Multiclass Unet) model is an innovative medical image segmentation framework that utilizes the feature pyramid to meet the demand for enhanced precision in medical image segmentation. The framework is based on the hidden hypothesis that the model can capture large objects more effectively when the receptive field expands and the encoder subnetwork layer deepens, and small objects and precise details when the compatible semantically rich feature maps from the decoder sub-network are fused with the excellent quality feature maps from the encoder sub-network after they have been progressively enriched. The suggested model concatenates encoder feature maps with upsampled decoder representations at several levels (X1 to X5) in order to fuse encoder-decoder features through skip connections. Prior to being sent to the last softmax layers for segmentation and classification, each fusion step uses convolutional transformations (CBL blocks) to enhance the features.

IHSnet makes use of a fully convolutional network framework, which efficiently learns and infers from inputs of any size to produce outputs of the same size. The framework’s main components can be changed at will to accommodate various scenarios like high precision, great efficiency, or a minimal network size, for instance. It is applicable to segment things with varying classes. Large objects, tiny objects, and precise information can all be captured more successfully by the architecture.

**Fig. 6:**
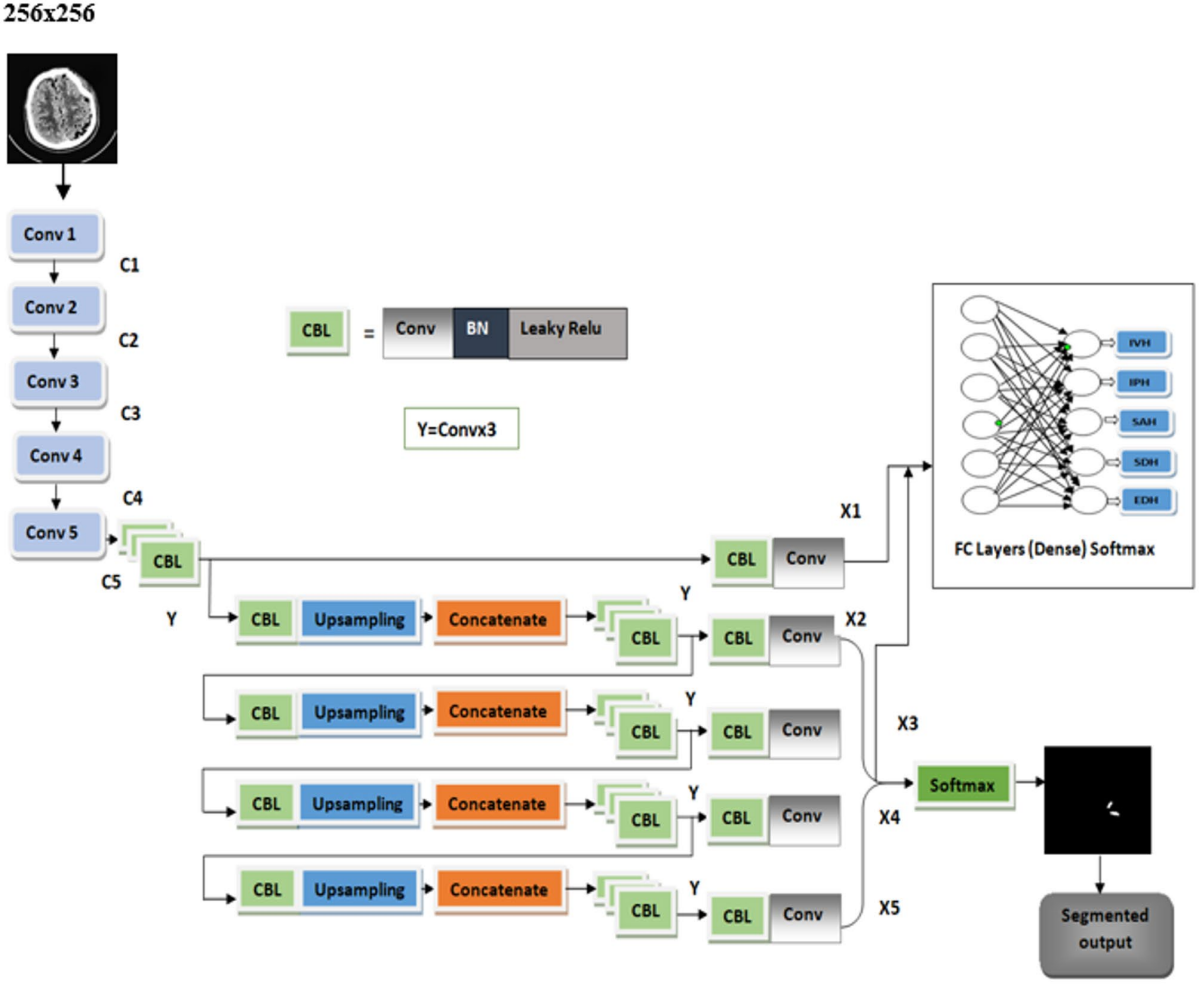
Proposed MUNet architecture.

An outline of the suggested architectural design is presented in Fig. 6. Then input image of dimension 256 × 256 is given as input. Next, the backbone extracts the features from the input image, which is the subnetwork of encoders. The resultant feature maps are described as (C1 − C5), and the backbone is composed of up of 5 convolution blocks Conv1-Conv5. The sizes of C1, C2, C3, C4 and C5 are 128 × 128 × 64, 64 × 64 × 128, 32 × 32 × 256, 16 × 16 × 512, and 8 × 8 × 1024, respectively, based on our assumption that the input image is 256 × 256 × 3.The special convolution layer replace the position of the max-pooling layer. The kernel size is assigned to 3 × 3, padding set to 0, and stride set to 2, these unique convolution layers ensure that the height, width of feature maps are divided in half and the channels count doubles as the convolution blocks increases.C5 is fed into two different network branches: one is used to predict the input image categories, while the other is used to create segmentation masks.

The input image is convolved over by the convolutional layers kernel in each channel, resulting in a complete reduction of the spatial size and dimension. It is essential to obtain the feature map after convolving the input image in its original size without losing any information in order to preserve the information and produce greater accuracy outcomes in the classification process. The information will be saved by padding the input picture matrix by 0 to store the intensity values. This aids the kernel in convolution around the image, producing features that precisely match the original input matrix’s spatial size. Strides are used to control the kernel convolving’s movement over the input image.

The kernel moves one pixel at a time if stride equals 1. If the number is 2, the image should be moved forward by 2 pixels at a time. The decoder sub-network comprises three blocks, namely 3DBL (a convolution layer, layer of batch normalization and leaky relu) that shortens the count of feature channels, and a feature map upsampling process, after which the appropriate C from the encoder sub-network is concatenated at each step. The feature maps, (C1 − C5) are upsampled and then concatenated (Conv1 − Conv5). Prior to the sigmoid function, we add a 1 × 1 convolution layer for every upsampling stage. This helps to minimize dimension, introduce nonlinearity, cross-channel information, and speed up the computation. One-by-one convolution layer and batch normalization are used to increase deep network training and address the issue known as internal covariate shift. We acquire a deep feature representation (C5) by means of a sequence of convolutional layers (Conv1–Conv5). Then, using skip connections, this representation is gradually upsampled and concatenated with matching encoder features. The segmentation output is generated by passing these decoder outputs (X2–X5) through convolutional blocks and a softmax layer, whereas the feature maps from various decoder levels are compressed then fed into fully linked layers which are followed by a softmax activation which helps in multiclass classification of hemorrhages concurrently with the segmentation process. As a result, our multi-task learning strategy enhances performance by enabling both segmentation and classification by making use of common features in the images. The algorithm given below illustrates the pseudocode.

#### Convolutional networks with batch normalization

Batch Normalization is suitable for any combination of network functions. Here, we focus on transformations that comprise of an element-wise nonlinearity and an affine transformation:

1 $$\:\:\:\:\:\:\:\:\:\:\:\:\:\:\:\:\:\:\:\:\:\:\:\:\:\:\:\:\:\:\:\:\:\:\:\:\:\:\:\:\:\:\:\:\:\:\:\:\:\:\:\:\:\:\:\:\:\:\:\:\:\:\:\:\:\:\:\:\:\:\:\:\:\:\:\:\:\:\:\:\:\:\:\:\:\:\:\:y=h({X}_{u}+b)$$

Where $\:X$ and $\:bare$the model’s learnt parameters, while $\:h(.)$represents a nonlinearity like the sigmoid or ReLU. Convolutional and fully-connected layers are covered by this formulation. Just prior to the nonlinearity, we apply the BN transform by normalizing $\:x={X}_{u}+b.$ Although $\:u$ is probably the output of another nonlinearity, its distribution shape is likely to vary during training, so we may have also normalized the layer inputs $\:u$. However, doing so would not have eliminated the covariate shift. $\:{X}_{u}+b\:$on the other hand, is more likely to yield activations with a stable distribution since it has a symmetric, non-sparse distribution, which normalizes it to be “more Gaussian”. We can disregard bias $\:b\:$because the mean subtraction that follows will balance out its effect, as we have normalized $\:{X}_{u}+b.$

In order to normalize diverse parts of the same feature map uniformly at various locations, we also want the normalization for convolutional layers to adhere to the convolutional property. In order to accomplish this, we jointly normalize each activation across all of them in a batch. For every activation in a given feature map, the BN transform applies the same linear transformation.

#### Higher learning rates with batch normalization

In conventional deep networks, an excessively high learning rate can cause gradients to inflate, disappear, or become trapped in unfavourable local minima. These problems are mitigated by batch normalization. It also keeps the training from becoming trapped in the saturated regimes of nonlinearities by leveling activations across the network, preventing modest parameter changes from amplifying into larger and suboptimal changes in activations in gradients. Additionally, batch normalization strengthens the training’s resistance to parameter scale changes. Large learning rates typically cause layer parameter scales to grow, amplifying the gradient during backpropagation and ultimately resulting in the explosion of the model. The scale of its characteristics has no bearing on propagation through a layer. On the other hand, backpropagation across a layer is independent of the parameter scale when using Batch Normalization. After all, given a scalar $\:a$,

2 $$\:BN\left({X}_{u}\right)=BN\left(\left(aX\right)u\right)$$

Therefore, the gradient is not impacted by the scale. Furthermore, batch normalization helps in model regularization and stabilizes the parameter growth since bigger weights result in lower gradients. The following are some benefits of using batch normalization:

- It prevents the issue of the vanishing gradient from developing.
- The insufficient weight initialization can be successfully managed by using it.
- The time needed for network convergence is greatly shortened (very helpful for large-scale datasets).
- It reduces training dependence across hyper-parameters.
- Over-fitting has less effect on regularization, the likelihood of it occurring is very low.

#### Leaky RELU

Generally deep learning and the rise of deep architectures have also brought attention to another shortcoming of the two conventional activation functions. In back-propagation, their limited output limited the derivatives’ dissipation when the network was deep. Stated alternatively, this means that deeper layers learned throughout training and observed virtually no changes to their weights. The vanishing gradient problem is reason for this occurrence. The rectified linear unit (ReLU) was introduced to partially address the challenges associated with computational calculation and deep learning.

3 $$y~ = \max \left\{ {~0,x} \right\} = x~\left| x \right\rangle 0K_{i} ~$$

ReLU is computationally efficient while achieving excellent performance. Gradients in back-propagation have a greater probability of reaching deeper layers since it does not impose restrictions on positive inputs, which allows for deeper layer learning. Furthermore, backpropagation learning simplifies the gradient to a constant multiplication calculation, which is significantly more efficient in computation. Thus, using neural networks for learning and inference have entered a completely new phase that has dominated research over the past ten years.

Major limitation of the ReLU is that it deactivates numerous neurons during training because it does not respond to negative inputs. This can be thought of as a vanishing gradient issue for negative values. Additionally, the learnable parameter influences both positive and negative values, which is the leaky Relu’s major strength. Its primary application is in the resolution of the Dying ReLU issue. Usually, it is set to an extremely low number like 0.001.

#### Loss function

Loss functions are an essential component of every deep learning model because they set a benchmark against which the MUNet network’s performance is evaluated and because the model learns its weight parameters by minimizing a selected loss function. The main goal of this experiment is to access the performance of loss functions with focal and, Dice loss. In order to compare the predicted class probability with the actual class desired output, the cross-entropy is used. The probability’s variance from the real expected value suffers a loss which is computed that penalizes the probability. The logarithmic penalty results in a large score when the differences are substantial and near to 1, and a small score when the discrepancies are tiny and go toward 0. In this case every training image input is labelled with a ground truth segmentation mask target $\:w$ and a ground truth class $\:v$. We employ a multi-task loss of $\:L$ on for every input image in order to collectively train for both mask segmentation and classification.

4 $$\:\:\:\:\:\:\:\:\:\:\:\:\:\:\:\:\:\:\:\:\:\:\:\:\:\:\:\:\:\:\:\:\:\:\:\:\:\:\:\:\:\:\:\:\:\:\:\:\:\:\:\:\:\:\:\:\:\:\:\:\:\:\:\:\:\:\:\:\:\:\:\:\:\:\:\:\:\:\:L={L}_{fl}+\lambda\:{L}_{ms}$$

Where the true class $\:v$, is focal loss expressed as $\:{L}_{fl}.\lambda\:\:$is the balance coefficient. The output of the segmentation mask branches characterizes the second task loss,$\:{\:L}_{ms}$. To evaluate the quality of image segmentation, we usually use the Dice coefficient, a similarity metric linked to the Jaccard index. Across the four segmentation mask branches’ output, the second task loss,$\:{\:L}_{ms}$ is defined. The following is the definition of the coefficient for segmentation output $\:{w}^{{\prime\:}}$ and target $\:w$ with the loss of Dice coefficient is $\:1-{D}_{i}$.

5 $$\:{\:\:\:\:\:\:\:\:\:\:\:\:\:\:\:\:\:\:\:\:\:\:\:\:\:\:\:\:\:\:\:\:\:\:\:\:\:\:\:\:\:\:\:\:\:\:\:\:\:\:\:\:\:\:\:\:\:\:\:\:\:\:\:\:\:\:\:\:\:\:\:\:\:\:\:\:\:D}_{i}\left({w}^{{\prime\:}},w\right)=\frac{2\left|w\cap\:{w}^{{\prime\:}}\right|}{\left|w\right|+\left|{w}^{{\prime\:}}\right|}$$

#### Fully connected layers

In neural networks, fully linked layers serve as the most versatile layer and are utilized in nearly every kind of architecture. In a completely connected layer, every node is linked to every other node in the layer before and after it. A fully linked layer’s primary goal is to modify the feature space to increase the problem’s malleability. The number of dimensions may rise, decrease, or remain constant throughout this transformation process. The new dimensions in each instance are linear combinations of the dimensions from the layer before. Then, non-linearity is added to the extra dimensions with the aid of an activation function.

Any type of interaction between the input variables is made feasible by FC layers. With enough depth and width, fully connected layers can theoretically learn any function because of this structure-agnostic method. Convolutional and recurrent layers are examples of more specialized layers that researchers have created in order to address this issue. In essence, these layers use the inductive bias based on the spatial structures of particular data types, including text, images, videos, etc. In this work, the fully connected layers help to classify ICH.

### Training process of the proposed model IHSNet

The algorithm shows how the training phase is carried out in MUNet which involves parameters like δ1 for training set and δ2 for the testing set whereas an iteration phase α is assigned for input layer of the CNN which helps in connecting the resultant layers C1 with C2 and C2 with C3 and C3 with C4 and finally C4 with C5. The parameters of MUNet were initialized as per the Table 5 and batch size is assigned and loss functions are computed and the remaining entire process is depicted in algorithm 1.

**Algorithm 1:**
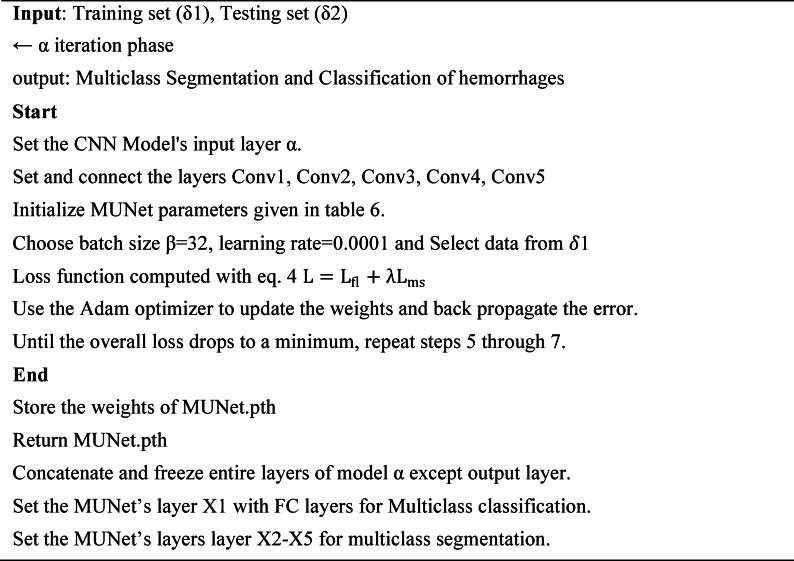
MUNet training process.

**Table 5:** Hyperparameters (Training).

Hyperparameters	Specification	Rationale
Input image size	256 × 256	Images resized to 256 × 256 maintain enough detail for segmentation while lowering computing costs.
Learning rate	0.0001	A lower learning rate is essential for segmentation which guarantees stable convergence and avoids optimal weights from being overshot.
Drop-in learning rate factor	0.5	When validation performance stalls, this cuts the learning rate in half, assisting the model in optimizing its weights for subsequent epochs.
Epochs	60	Enough training is possible with 60 epochs without overfitting. If training does not improve, early halting can be utilized to cease it.
Batch size	32	Memory efficiency and gradient stability are balanced by a batch size of 32, particularly when multi-GPU training is being used.
Optimizer	Adam	Adam eliminates vanishing/exploding gradients and adjusts the learning rate; it is frequently employed in segmentation problems.
Loss	Dice loss	Compared to more conventional losses like cross-entropy, dice loss is better for segmentation since it directly optimizes for region overlap.
Environment	MultiGPU	Training is accelerated by using multiple GPUs, which also allows for bigger batch sizes for consistent gradient updates.

### Performance metrics

The competency of the proposed work is demonstrated using a range of metrics, such as Accuracy, Specificity, Sensitivity, Recall, Precision, F1 score, Dice coefficient, and IoU. It is clear from the simulation findings that the recommended strategy offers a dependable way to categorize and classify different types of intracranial hemorrhages. The terms “true positives,” “true negatives,” “false positives,” and “false negatives” are shortened to TP, TN, FP and FN respectively, in this study. These metrics are looked at in order to evaluate the experimental results.

### Confusion matrix

The application of a confusion matrix is a crucial component of our performance assessment. False positive (FP), false negative (FN), true positive (TP), and true negative (TN) counts predictions is displayed in this tabular layout, which enables a concise summary of the classification model’s effectiveness with the aid of the confusion matrix. Table 6 is the standard format in which the confusion matrix is displayed.

**Table 6:** Confusion matrix.

Actual	Predicted	
	True positive	False negative
	False positive	True negative

### Accuracy

The outcome of classification is assessed by the accuracy (ACC) metric, which is denoted by

6 $$\:\:\:\:\:\:\:\:\:\:\:\:\:\:\:\:\:\:\:\:\:\:\:\:\:\:\:\:\:\:\:\:\:\:\:\:\:\:\:\:\:\:\:\:\:\:\:\:\:\:\:\:\:\:\:\:\:\:\:\:\:\:\:\:\:\:\:\:\:ACC=\frac{TP+TN}{TP+FP+TN+FN}$$

### Precision

The precision metric (PREC) is characterized as the proportion of correctly projected positive classifications for every positive prediction. It can be obtained numerically by

7 $$\:\:\:\:\:\:\:\:\:\:\:\:\:\:\:\:\:\:\:\:\:\:\:\:\:\:\:\:\:\:\:\:\:\:\:\:\:\:\:\:\:\:\:\:\:\:\:\:\:\:\:\:\:\:\:\:\:\:\:\:\:\:\:\:\:\:\:\:\:\:\:PREC=\frac{TP}{TP+FP}$$

### Specificity

Specificity (True Negative Rate) is a statistic that can be used to assess the proportion of correctly classified negative instances to entire negative cases.

8 $$\:\:\:\:\:\:\:\:\:\:\:\:\:\:\:\:\:\:\:\:\:\:\:\:\:\:\:\:\:\:\:\:\:\:\:\:\:\:\:\:\:\:\:\:\:\:\:\:\:\:\:\:\:\:\:\:\:\:\:\:\:\:\:\:\:\:\:\:\:\:\:\:\:\:SPEC=\frac{TN}{TN+FP}$$

### Sensitivity

Sensitivity (SEN) or True Positive Rate (TPR) is a statistic that can be used to assess the proportion of correctly classified positive instances to all positive cases.

9 $$\:\:\:\:\:\:\:\:\:\:\:\:\:\:\:\:\:\:\:\:\:\:\:\:\:\:\:\:\:\:\:\:\:\:\:\:\:\:\:\:\:\:\:\:\:\:\:\:\:\:\:\:\:\:\:\:\:\:\:\:\:\:\:\:\:\:\:\:\:\:\:\:\:SEN=\frac{TP}{TP+FN}$$

### F1 score

PREC and recall’s harmonic mean is calculated mathematically with the F1 score.

10 $$\:\:\:\:\:\:\:\:\:\:\:\:\:\:\:\:\:\:\:\:\:\:\:\:\:\:\:\:\:\:\:\:\:\:\:\:\:\:\:\:\:\:\:\:\:\:\:\:\:\:\:\:\:\:\:\:\:\:\:\:\:\:\:\:\:\:\:\:\:\:F1\:Score=\frac{2.TP}{2.TP+FP+FN}$$

### Dice similarity coefficient

The main metric of evaluation used to assess how close the prediction and ground truth are is the Dice Similarity Coefficient (Dice), which is a widely used metric to assess segmentation where |A| is the actual volume of an image, |B| is the projected volume of an image.

11 $$\:\:\:\:\:\:\:\:\:\:\:\:\:\:\:\:\:\:\:\:\:\:\:\:\:\:\:\:\:\:\:\:\:\:\:\:\:\:\:\:\:\:\:\:\:\:\:\:\:\:\:\:\:\:\:\:\:\:\:\:\:\:DSC\left(A,B\right)=\frac{2\left|A\cap\:B\right|}{\left|A\right|+\left|B\right|}$$

### Intersection over union

An essential segmentation metric which determines whether a prediction about an object is precise or not is an intersection over union, commonly called Jaccard index.

12 $$\:\:\:\:\:\:\:\:\:\:\:\:\:\:\:\:\:\:\:\:\:\:\:\:\:\:\:\:\:\:\:\:\:\:\:\:\:\:\:\:\:\:\:\:\:\:\:\:\:\:\:\:\:\:\:\:\:\:\:\:\:\:\:\:\:\:\:\:\:\:\:\:\:\:\:\:\:\:\:\:\:\:\:IOU(A,B)=\frac{|A\cap\:B|}{|A\cup\:B|}$$

### AUC-ROC curve

The ability of the model to distinguish between positive and negative examples can be assessed using AUC-ROC measure. The false positive rate (specificity) at various categorization thresholds is contrasted with the true positive rate (recall) in the ROC curve. An identifier that represents the model’s capacity for discrimination is provided by the AUC-ROC, which covers the region under this curve. AUC-ROC values of 0.5 are indicative of a random or improper classifier, whereas classifiers that are perfect, ideally provide an AUC-ROC value closer to 1.

## Experimental results

This section describes the experimental design that we used for this study, which contains a thorough assessment of our MUNet model, along with in-depth analyses of several evaluation metrics, such as recall, accuracy, precision, F1-score, Dice coefficient and IoU. We also show the visual illustration of receiver operating characteristic (ROC) curves, loss curves, and confusion matrices.

### Simulation environment

In order to train our model IHSNet, we used NVIDIA Apex with a single NVIDIA P100 GPU that was offered on Kaggle. For better training and to shorten training times, all images were downsampled to 256 × 256 pixels. Model parameters are optimized during the training phase by using the optimizer (Adam). The first learning rate that we set was 0.0001. The model that performs the best is selected, and the maximum training epoch is set to 60. The Table 5 displays the training hyperparameters of the proposed IHSNet.

The training process is based on the choice of batches and the initial weights of the models, which makes the experiments reproducible and gives an accurate depiction of the improvements made by changing the training options and hyperparameters. It is essential to explain a few of the rows in Table 5 before going into the hyperparameter tuning process. These rows include batch size, number of epochs and other terms whose names might be confusing given the terminology used frequently in DL papers. To estimate the moments in an adaptable manner, which stabilizes convergence, we choose the Adam optimizer with 0.0001 learning rate. We include a Drop-in Learning Rate Factor of 0.5 to avoid overfitting, which dynamically lowers the rate of learning when the validation loss stagnates. Furthermore, our design incorporates Batch Normalization into convolutional layers to mitigate internal covariate changes by regularizing feature distributions. To improve generalization, dropout layers are incorporated. When combined, these strategies provide consistent training and enhanced segmentation performance.

### Training and testing the IHSNet model

Accuracy validation, model loss is used to assess the model’s training procedures. The rounds of iterations utilized in training for extracting features are known as the epochs which forward it to the subsequent learning layers. The model is not acquiring learning and exhibits overfitting if the accuracy of the model drops and the loss increases during the training period. The indicator shows the model is learning when accuracy rises and loss falls. The input images have dimensions of 256 × 256. We employed the Adam optimizer with 32 allotted as the size of the batch and 0.0001 alloted as learning rate throughout the experiment.

**Fig. 7:**
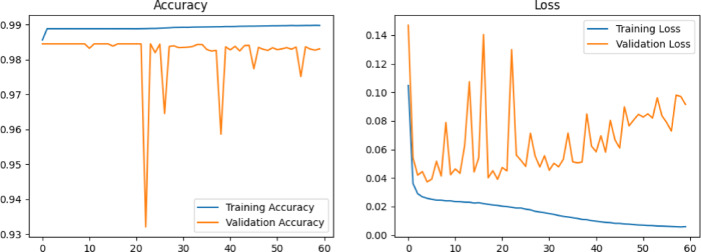
Model training and validation curves in accuracy & loss terms.

**Fig. 8:**
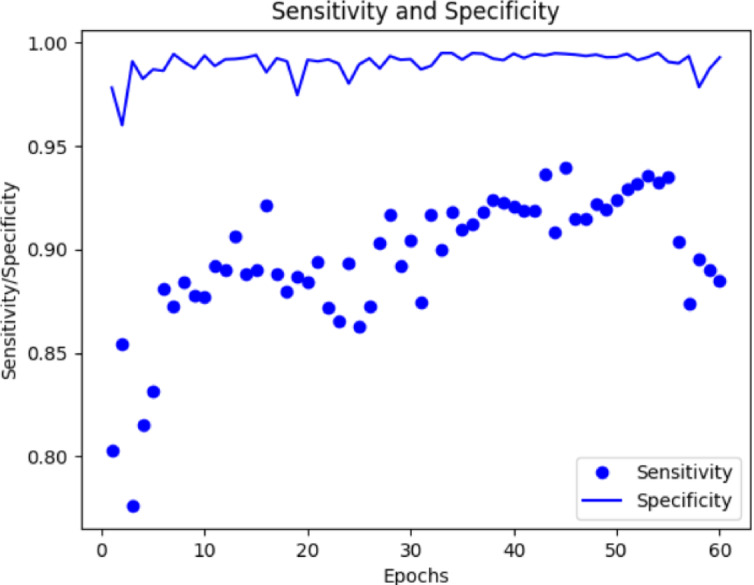
Sensitivity vs. Specificity.

Regarding the train and test data, Fig. 7 shows the accuracy and loss of the suggested model and Fig. 8. depicts the sensitivity and specificity curves. Using the TensorFlow library, the experiment’s practical part was carried out in the Kaggle environment. The proposed model IHSNet was allotted sixty epochs to train and finish where a total of 1960 images (training) and 71 images (testing). The model’s derived experimental results showed reasonable losses and accuracy for both training and validation. The training and validation accuracy were 98.53 and 95.67, respectively, with sensitivity and specificity reaching 0.94 and 0.98, respectively for epochs count of 60.

### Resizing and CLAHE output

**Fig. 9:**
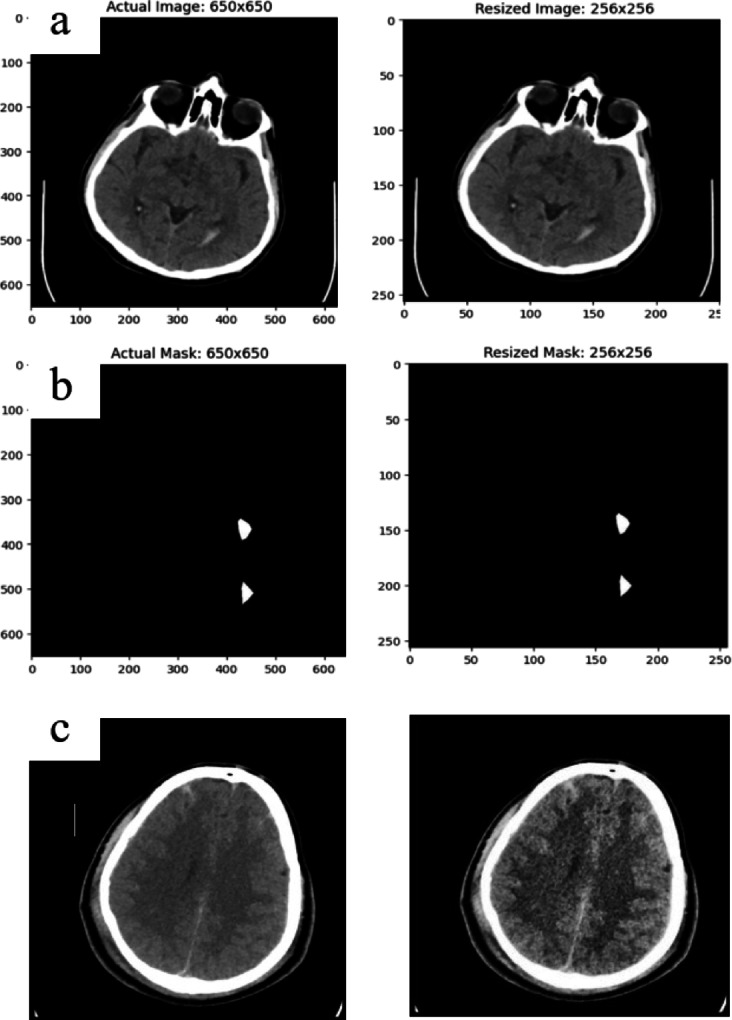
(**a**) Actual image vs. resized image (**b**) Actual mask vs. resized mask (**c**) Actual image vs. CLAHE implemented image.

The deep learning models must be trained with images of the same dimensions. The process of learning is accelerated and the possibility of overfitting is decreased by shrinking images to the appropriate sizes. One of the difficult aspects of scaling photos is the loss of data, which degrades both the accuracy rate and the model’s performance. The images and masks were shown in Fig. 9 involving the pre and post resizing process, as well as the CLAHE applied image is also compared with the actual image.

### Segmentation output

**Fig. 10:**
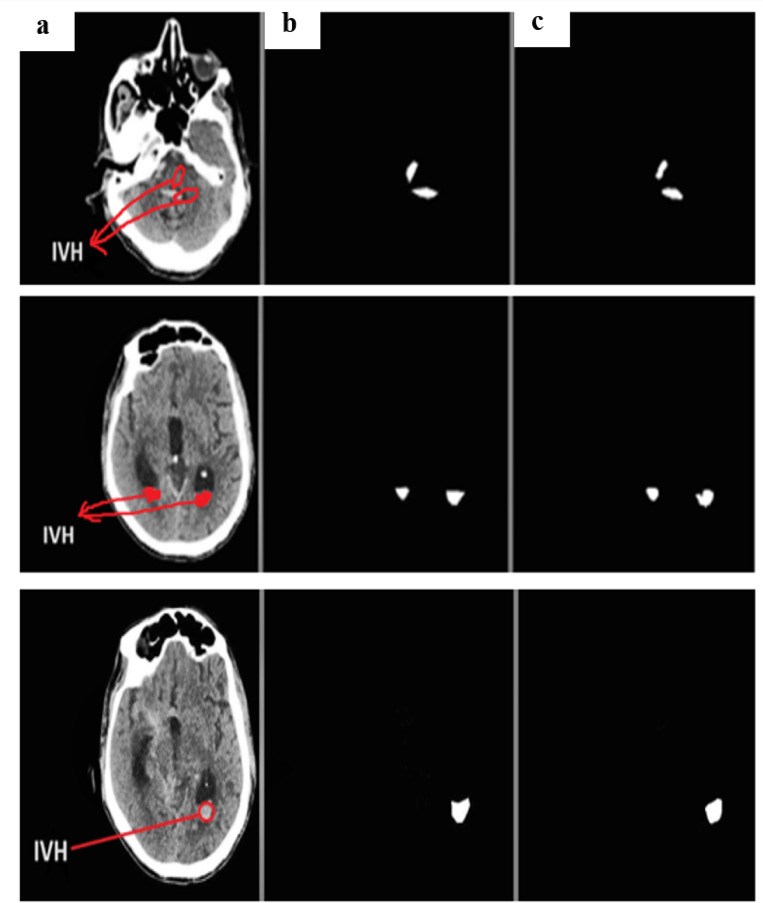
(**a**) IVH input image (**b**) actual mask (**c**) predicted mask.

**Fig. 11:**
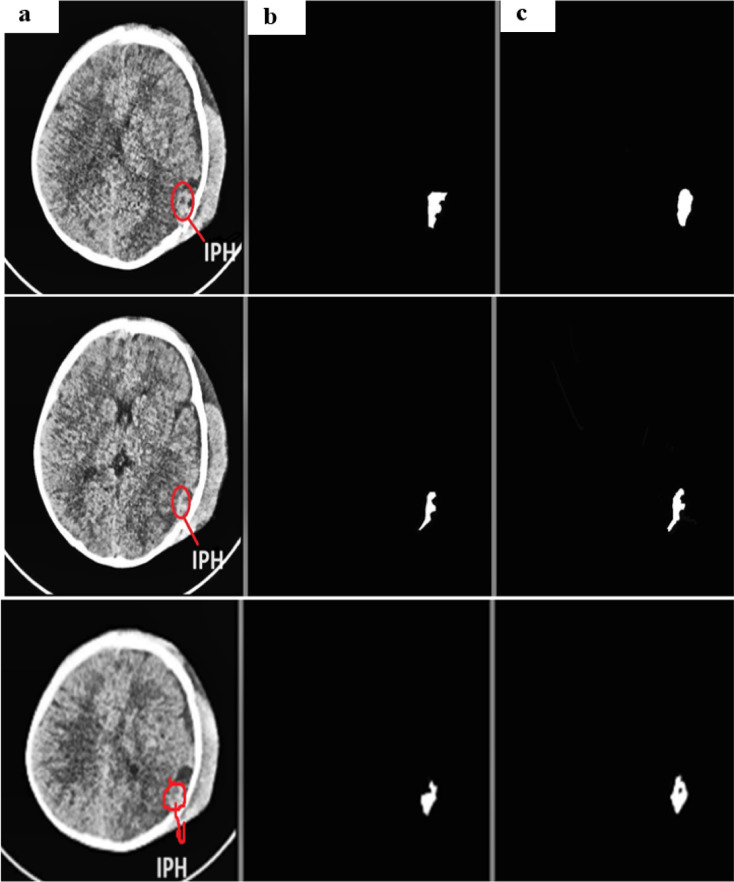
(**a**) IPH input image (**b**) actual mask (**c**) predicted mask.

**Fig. 12:**
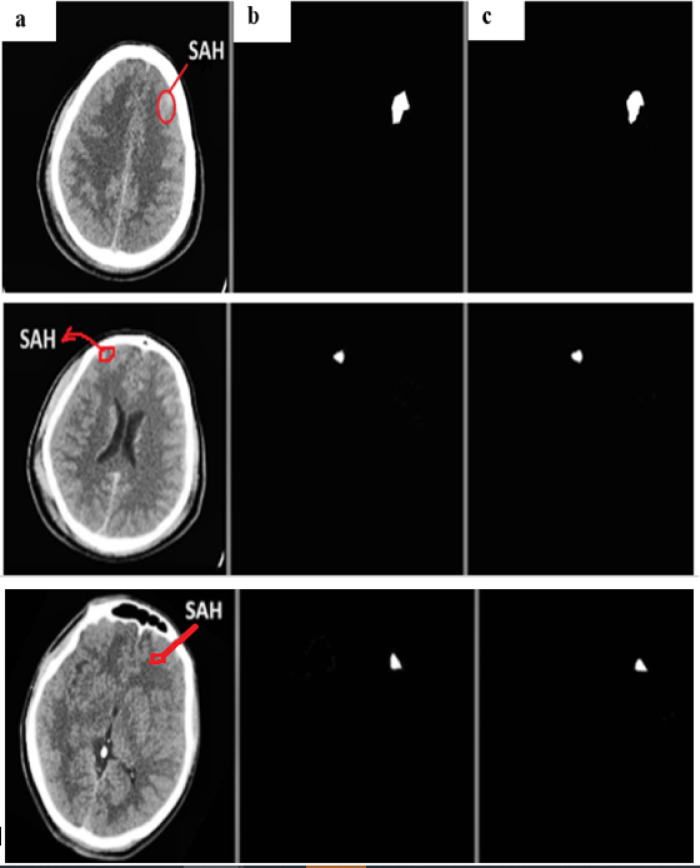
(**a**) SAH input image (**b**) actual mask (**c**) predicted mask.

**Fig. 13:**
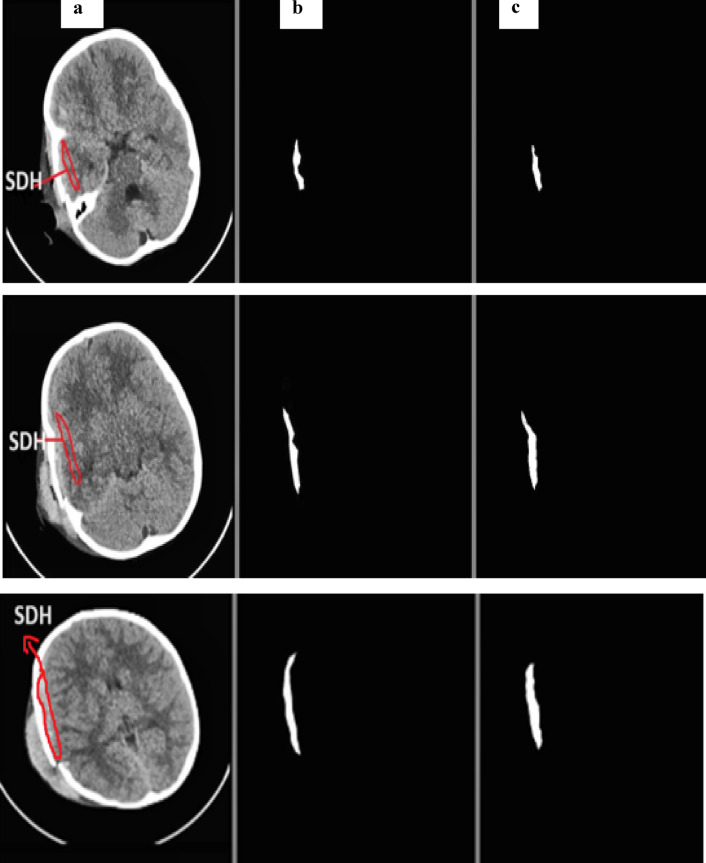
(**a**) SDH input image (**b**) actual mask (**c**) predicted mask.

**Fig. 14:**
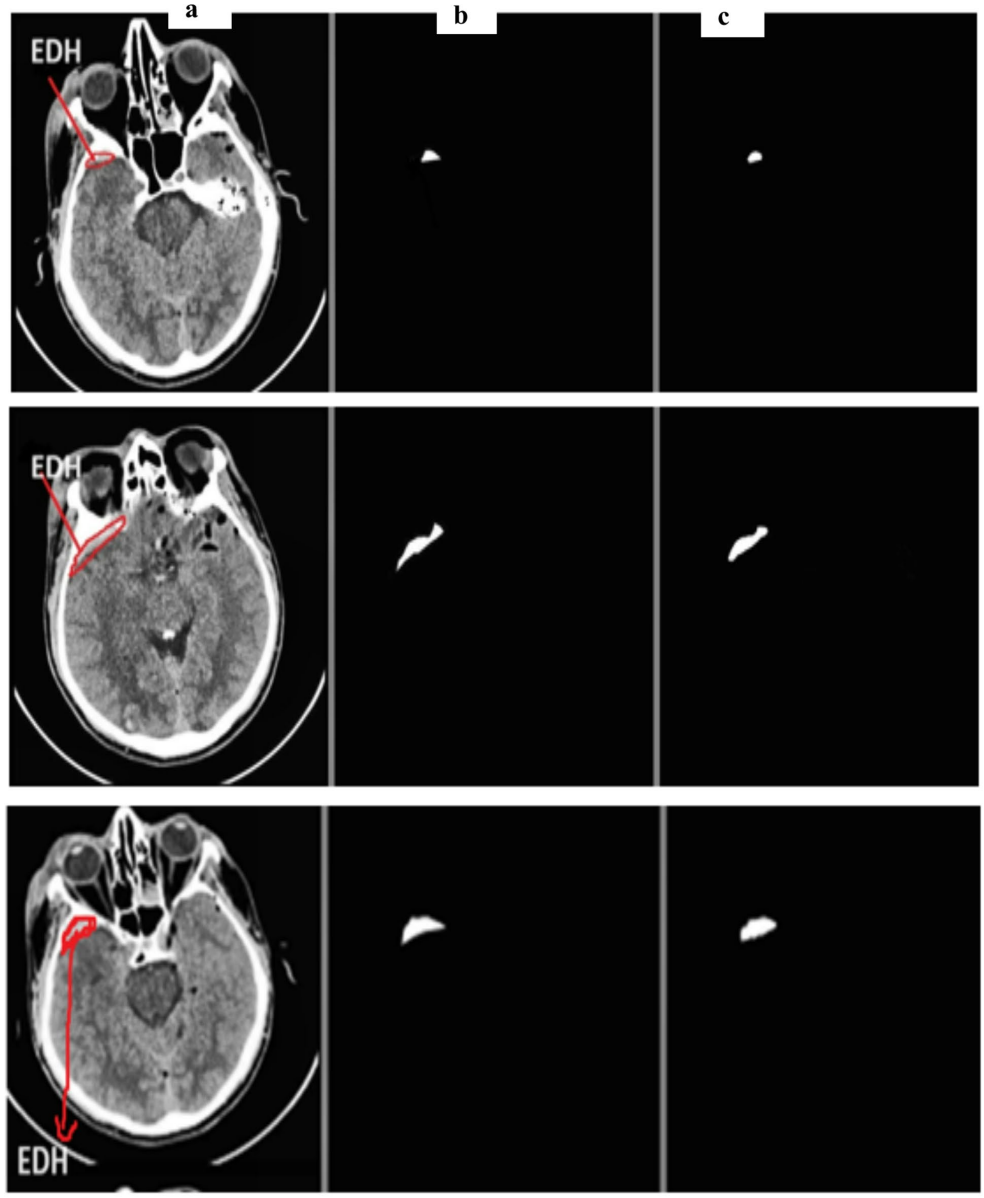
(**a**) EDH input image (**b**) actual mask (**c**) predicted mask.

Using the physionet dataset, the segmentation method of MUNet was experimentally evaluated for five classes. The segmentation images and their expected masks for each class were compared and reviewed, as shown below in the Figs. 10, 11, 12, 13, 14. In the experimental investigation, we segmented the multiclass hemorrhages and assessed the model’s effectiveness using the Dice coefficient and IOU. The model is trained with a ratio of 80:20 for the training and test sets, respectively, and the segmentation performance is assessed using the available dataset. A total of 71 images are set aside for testing and 1960 images for training. The parameters of batch size assigned as 32, epochs count as 60, and the learning rate to 0.0001. Two widely used metrics, Jaccard index and the dice coefficient are employed in the evaluation which is regularly used for segmentation trials. An addition metric intraclass correlation coefficient was evaluated with a value of 0.87.

Figure 10 depicts the segmented samples of IVH from the Patient id-80, where the representations are given as (a) IVH input image (b) actual mask (c) predicted mask.

Figure 11 depicts the segmented samples of IPH from the Patient id-50, where the representations are given as (a) IPH input image (b) actual mask (c) predicted mask.

Figure 12 depicts the segmented samples of SAH from the Patient id-76, where the representations are given as (a) SAH input image (b) actual mask (c) predicted mask.

Figure 13 depicts the segmented samples of SDH from the Patient id-51, where the representations are given as (a) SDH input image (b) actual mask (c) predicted mask.

Figure 14 depicts the segmented samples of EDH from the Patient id-49, where the representations are mentioned as (a) EDH input image (b) actual mask (c) predicted mask.

The Table 7 shows the dice coefficient, IoU and intra class coefficient outcome of the proposed MUNet model during ICH segmentation.

**Table 7:** Performance metrics of ICH segmentation.

ICH Type	Dice coefficient	IOU	Mean dice coefficient (Testing)	Mean IoU (Testing)	Mean intra class coefficient
IVH	0.77	0.67	0.79	0.68	0.87
IPH	0.80	0.71			
SAH	0.92	0.82			
SDH	0.64	0.51			
EDH	0.84	0.69			

The ICH sub-type findings indicated that MUNet providing the EDH, SDH, IVH, IPH and SAH segmentation had dice scores of 0.84, 0.64, 0.77, 0.80 and 0.92 in that order respectively. The proposed segmentation approach achieved a mean Dice coefficient of 0.94 for the training dataset and 0.79 for the test dataset. The training and testing data were 0.80, 0.68 respectively for the IoU. To further illustrate the effectiveness of IHSNet, the segmentation results are displayed with an additional metric called interclass class coefficient which gave a satisfactory mean value of 0.87.

### Ablation study

Insightful ablation studies are also provided for the model’s continued comprehension. Furthermore, we discover that the use of feature pyramid network and SMOTE technique are crucial for linking hemorrhagic characteristics, which is important for the diagnosis procedure. We present ablation studies for the two suggested ways in Table 8.

**Table 8:** Ablation experiments.

Method/evaluation	Metrics	IVH	IPH	SAH	SDH	EDH
Munet + SMOTE	Dice	0.59	0.64	0.70	0.50	0.69
	IoU	0.45	0.50	0.59	0.35	0.56
Munet + FPN + SMOTE (Proposed)	Dice	0.77	0.80	0.92	0.64	0.84
	IoU	0.67	0.71	0.82	0.51	0.69
Munet + FPN	Dice	0.45	0.57	0.52	0.49	0.68
	IoU	0.34	0.42	0.37	0.35	0.55

Table 8 shows that both feature pyramid and SMOTE method adding resulted in minor increases, with notable gains in dice and iou values. When SMOTE is the only network technique used and the feature pyramid network is omitted, the performance deteriorates. Though the results produced while excluding the feature pyramid network and including SMOTE along with Munet were satisfying but the integration of feature pyramid network along with MUnet and SMOTE produced more promising results compared to the other cases in ablation study. Though there is an issue of training and inference time-consumption, especially on large images we tried with only 200 images of 512 × 512 resolution and tested with 71 images of the same resolution which gave overall dice and iou of 0.73 and 0.62, but due to time constraint and more computational resource consumption, entire images count of over 2000 were not trained and tested.

#### Classification output

**Fig. 15:**
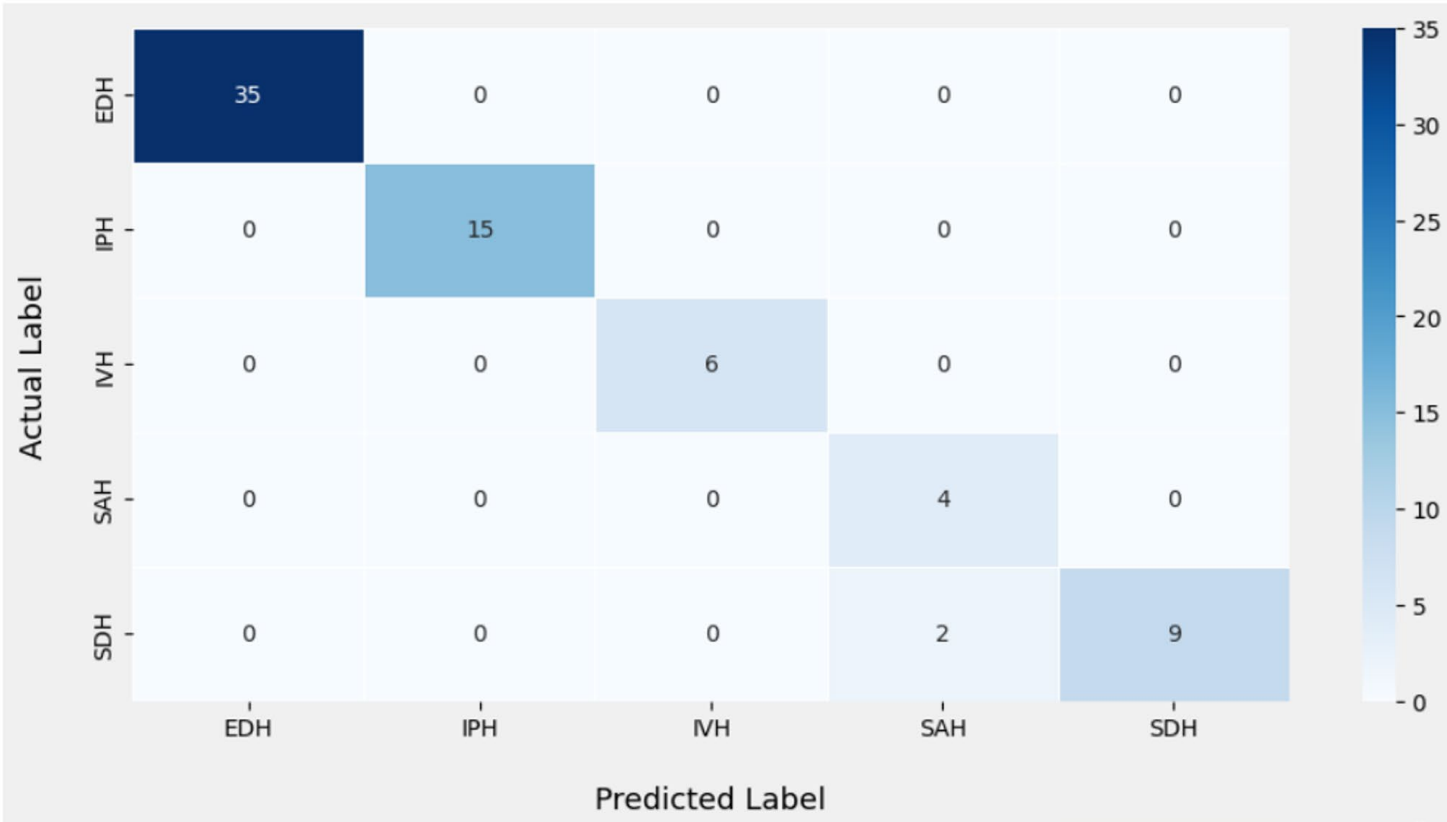
Confusion matrix for multiclass hemorrhage classification.

The following section evaluates the categorization outcomes of the multiclass hemorrhages in brain images according to their specific type. The MUNet method’s confusion matrices on 20% test data, which demonstrate the distinct classifications of five classes, are displayed in Fig. 15. The true class and the expected class are the two categories that are noted in the confusion matrix shown in Fig. 15, which also provides the metrics like precision, recall and f1 score which achieved a training accuracy of 99.02% and testing accuracy of 98.71%. The suggested model’s average classification accuracy is generally higher and more satisfactory.

This categorization has made it possible to identify the methods by which the multiclass hemorrhages were accurately predicted. It is evident that the suggested approach yields the fewest erroneous estimates. The Table 9 depicts the metrics f1 score, accuracy, precision and recall for the multiclass hemorrhage classification. According to Table 9, the precision values stand at 1 for IVH, IPH, EDH and SDH except SAH which scored a value of 0.67. Similarly, the recall values stand at 1 for EDH, SAH, IPH, IVH where the value of SDH is 0.82. F1 Scores stands at 1 for the types EDH, IPH and IVH scoring a peak value of 1, and SAH, SDH scored 0.80,0.90 respectively.

**Table 9:** Classification metrics of multiclass hemorrhages.

ICH Type	Accuracy	Precision	Recall	F1-Score
EDH	1.00	1.00	1.00	1.00
IPH	1.00	1.00	1.00	1.00
IVH	1.00	1.00	1.00	1.00
SAH	1.00	0.67	1.00	0.80
SDH	0.81	1.00	0.82	0.90

The accuracies yielded for multiclass hemorrhages classification are remarkable for all the types that scored 1, Except SDH scoring 0.81 accuracy.

**Fig. 16:**
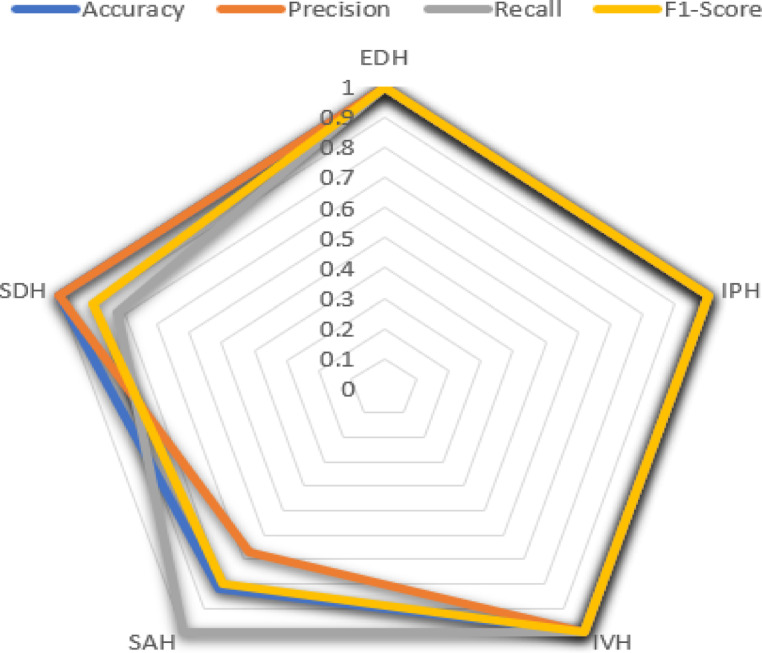
Precision, recall, F1 -score chart.

The diagram in Fig. 16 depicts the values in chart view, produced on the metrics Recall, Precision and F1-Score with various colours for identification.

**Fig. 17:**
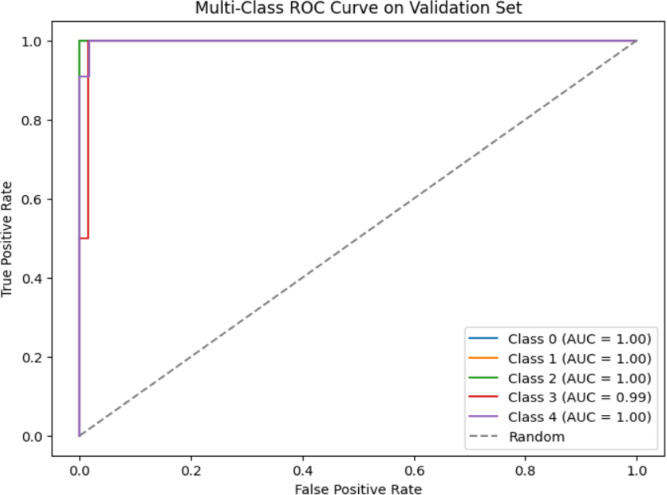
Multiclass ROC curve on validation dataset.

We completed the categorization and produced ROC curves for including the ranges of uncertainty for each assessment indication of multiclass hemorrhages, as shown in Fig. 17. The baseline, which is represented by the ROC curve, which shows for various multiclass should be noted. We witnessed the curve with variance by taking into account each of these curves when we calculated the average Area Under the Curve (AUC) for each class then showed in Fig. 17. The generated data offer perceptions into the factors determining the classifier’s output.

Figure 17 depicts that ROC curves were plotted for the subdural, subarachnoid, intraventricular, intraparenchymal, and epidural. Epidurals (Class 0), intraparenchymal (Class 1), intraventricular (Class 2) and Subdural (Class 4) possess a region below the curve of 1 whereas subarachnoid (Class 3) and possess a region below the curve of 0.99.

## Discussion

This study aims to validate that the proposed IHSNet performs better in segmenting ICH and classifying its five ICH subtypes than the state-of-the-art deep learning approaches. The suggested deep learning model enhances the MUNet baseline model’s specificity, sensitivity, and accuracy in diagnosing ICH. Targeting the five ICH subtypes specifically, the suggested MUNet outperforms the other five subtypes in terms of DICE coefficient, precision, recall, f1 score when compared to current techniques, MUNet’s segmentation performance is noticeably better. When it comes to segmentation visualization, the findings of MUNet’s segmentation are more consistent with feature extraction.

MUNet decreased the possibility of mistakenly recognizing the skull as a bleeding point for SDH and EDH. Furthermore, in contrast to alternative deep learning techniques, our model exhibits greater sensitivity in identifying the bleeding location. Consequently, there may be opportunities for clinical application since the suggested MUNet can more successfully segregate various ICH region types from ICH patients’ CT scans. Only 318 ICH slices were obtained from our dataset because 36 out of the 82 patients had an ICH diagnosis. Additionally, even though the ICH classification has a large number of publicly accessible datasets like CQ500, RSNA, and others, there isn’t one for the ICH segmentation. Furthermore, regarding the classification and detection, several studies are proposed for ICH segmentation algorithms.

An unbiased evaluation of the different approaches is not possible, nevertheless, as many of these techniques were not validated due to the lack of their corresponding ICH masks. Therefore, it is necessary to have a dataset like Physionet which is more feasible for our work since it has corresponding masks of the images that can help with benchmarking and establishing the work. To prevent class-imbalance problems between the data, we used the SMOTE approach. The comparison in the performance of the proposed and existing approaches/models during the Segmentation, classification are shown in Tables 10 and 11 respectively.

**Table 10:** Comparison of the suggested and current approaches/models with segmentation metrics during testing.

Authors	Dataset	Methods	Dice	IoU
Emon et al.^61^	Physionet	ResUNet-CutMix	0.61	0.44
Vamsi^62^	Physionet	Lightweight deep learning-based neural network	0.729	-
Nie et al.^63^	Physionet	KP-SAM	0.774	0.597
Wang et al.^64^	Physionet	U-Net along withip + ml + CA + ms + MRHDC	0.717	0.596
Proposed	Physionet	MUNet	**0.79**	**0.68**

**Table 11:** Comparison of the suggested and current approaches/models in terms of classification metrics.

Authors	Dataset	Method	AUC					Spe	Sen	Pre	Accuracy
			EDH	IVH	SAH	IPH	SDH				
Alfaer et al.^53^	Physionet	AICH-FDLSI	-	-	-	0.886	0.910	0.983	0.886	0.910	0.977
Wang et al.^65^	Physionet	2D CNN + sequence models	0.953	0.985	0.962	0.916	0.983	0.914	0.916	-	-
Agarwal et al.^66^	Physionet	CA-ConvNeXt	0.91	0.94	0.97	0.97	0.87	-	0.97	0.87	-
Anupama et al.^67^	Physionet	GC-SDL	-	-	-	-	-	0.97	0.94	0.95	0.95
Nizarudeen et al.^68^	Physionet	ICH-DC-ResNet-DenseNet-XG Boost	-	-	-	-	-	0.972	0.98	0.97	0.97
Hilal et al.^69^	Physionet	IICHD-BS	-	-	-	-	-	0.99	0.968	0.965	0.986
Proposed	Physionet	MUNet	1.00	1.00	0.99	1.00	1.00	0.94	0.98	0.93	0.987

The Tables 10 and 11 demonstrated the superior results that the proposed IHSNet framework has provided for a multiclass segmentation and classification. Coursed characteristics to the detailed features have been integrated through the use of feature pyramid networks, which aid in the convolutional layers to “understand” and concurrently assess the better features across multiple levels. In simpler terms, the computation of features defined by five backbones was aided, subsequent to which a network branch is applied for classification with fully connected layers and four additional network branches for multiclass segmentation masks containing feature maps that were upsampled and then concatenated with the aid of CBL layers. With an average dice coefficient of 0.79 and 0.68 IoU, the suggested methodology successfully classified diseases into several classes with an accuracy of 1 and are satisfying. Though the Strengths of this model is Effective Feature Extraction uses CBL blocks (Conv-BN-Leaky ReLU) in a deep encoder-decoder architecture for enhanced feature extraction and learning. Skip connections and concatenation layers are used to maintain spatial information. The model becomes more flexible when dual-task learning is used for segmentation and classification. In contrast to transposed convolutions, effective upsampling lowers computing expenses.

The acquired data demonstrate that the suggested model outperforms the other models and methodologies listed in the table. The lowest accuracy among classification goes to SDH category which scored 81% accuracy and the reason for its lowest accuracy might be the location of subdural type is near the skull, which makes it difficult to recognize lesions when they initially emerge. This results in a low dice score of 0.64 for SDH. Furthermore, SDH performs a bit worse than the other categories due primarily to the fact that subdural hemorrhages frequently have an irregular shape and expand into the sulci.

In the case of IPH, IVH segmentation, the MUNet can more precisely characterize the interface between the two forms of bleeding at the ventricle when parenchymal hemorrhage breaks into it. The MUNet Net lowers the possibility of misreading a skull for bleeding in cases of dural types (SDH, EDH). Moreover, the suggested model segments then detect SAH diffusing in sulci more sensitively with a good and satisfactory dice coefficient score of 0.79, whereas overall classification has produced outstanding results with accuracy 98.71%. However, the purpose of the test data is only to measure its quality and it is not intended to be used for training, indicating that the network isn’t expected to learn anything from it. Instead, it is only supposed to be used to adjust parameters. Since this implies that testing metrics are usually lower than training metrics. The major drawback of the proposed work is lack of attention-based mechanisms to enhance feature focus and no any explicit self-attention convolutions were deployed to identify the long-range dependencies. More regularization techniques are required since fully connected layers contribute high parameters. Though the IHSnet outperforms the traditional Unet and other CNN based classifiers, since unlike traditional Unet, IHS net uses CBL blocks for better representation of features then the feature pyramid network aids in better upsampling and enhanced multiclass feature fusion and refinement. Finally, IHSnet displays its superiority over CNN based classifiers by performing both segmentation and classification in a unified pipeline by reducing the computational overhead. Unlike traditional CNN classifiers, IHSnet model helps to extract and preserve fine features and also minimizes the need of separate models for classification.

## Conclusion

The serious medical condition known as intracranial hemorrhage (ICH) has a very high fatality rate. Formulating therapy and surgical plans requires the segmentation, identification of CT images of patients suspected of having ICH. As significant as this problem is, there aren’t many reliable ICH segmentation methods that don’t require a medical expert. As such, developing reliable and precise techniques for extracting ICH regions from CT images is essential. Since deep learning algorithms have low resolutions and a significant degree of variability in hemorrhagic location, contrast, and form, they provide difficulties when it comes to segmenting intracranial hemorrhages from CT scans. We have learned vital information from our research into deep learning algorithms for ICH segmentation.

In this research, we suggested an approach based on deep learning for classification and segmentation using MUNet. Further, MUNet is a complete convolutional encoder-decoder network with skip-connection between the encoder and decoder, deployed on feature pyramids to help in predicting the masks by forecasting the bleeding regions. It can also be used to determine the multiple-branching architecture and the categories within the region of interest which fulfils the requirement for more precise segmentation. CLAHE technique helps in improving the contrast of the images which is a vital one in predicting the tiny regions of bleeding. The study’s data set is insufficient, so the SMOTE approach is used for solving data imbalance issues and 80% of the images is utilized for training and 20% used for testing, whereas SMOTE was only applied on training images and not applied for testing images. The fully connected layers in the MUNet play a vital role in classification of various types of haemorrhages along with the sigmoid function. When their accuracy rates are high enough, these models may be able to perform better than experts in the field, which would reduce the number of false-negative ICH detections. A patient with ICH may not survive if treatment is delayed. The interval between symptoms and diagnosis is very important. Thus, the use of these modern techniques can aid in reducing this time. When utilized in medical settings and hospitals, the developed hemorrhage detection model is expected to be of great assistance to physicians and other healthcare units.

Simpler image formats (such as PNG, JPEG, and NumPy arrays) are the main focus of libraries like TensorFlow, PyTorch, and scikit-learn. When utilizing NIfTI directly, the pipeline becomes more complex because preprocessing and additional libraries, such as NiBabel are needed to import and manage the data. NIfTI files are typically larger than compressed PNG or JPEG files. When working with huge datasets, this can cause considerable delays in data loading, training, and storage. On the other hand, Physionet is a common benchmark public dataset that includes cases with different patient demographics, anatomical locations, and severity. This improves our model’s applicability in actual clinical settings. Our dataset’s ground truth annotations were created and validated by experienced radiologists. By doing this, the data evaluation can be assured to conform to clinical standards. While the model does not introduce entirely new architectural concepts beyond existing U-Net enhancements, the novelty lies in the unique combination and integration of MUnet with techniques like FCN, feature pyramid, batch normalization to achieve superior segmentation performance. Our major focus is on optimizing segmentation accuracy and robustness for ICH segmentation and detection. We integrate fully connected layers, feature pyramid networks, and batch normalization to enhance feature extraction, multi-scale learning, and training stability. These refinements lead to improved performance over baseline U-Net models and makes our approach valuable for real-world clinical applications.

## Data Availability

The data will be provided based on the reasonable request to the corresponding author (Prakasam P).

## Abbreviations

ICH: Intracranial hemorrhage
CT: Computed tomography
DICOM: Digital imaging and communications in medicine
SAH: Subarachnoid hemorrhage
SDH: Subdural hemorrhage
IVH: Intraventricular hemorrhage
IPH: Intraparenchymal hemorrhage
EDH: Epidural hemorrhage
AI: Artificial intelligence
AUC: Area under the curve
FC: Fully connected
ROC: Receiver operating characteristic
AUC: Area under the curve
GPU: Graphical processing unit
ReLU: Rectified linear unit
BN: Batch normalization
DSC: Dice similarity coefficient
IOU: Intersection over union
